# Design and validation of an inducible and curable EvolvR system for directed evolution in *Corynebacterium glutamicum*

**DOI:** 10.3389/fbioe.2026.1827071

**Published:** 2026-05-26

**Authors:** Paul M. Will, Vanessa L. Göttl, Jan Seeger, Volker F. Wendisch, Nadja A. Henke

**Affiliations:** 1 Genetics of Prokaryotes, CeBiTec and Faculty of Biology, Bielefeld University, Bielefeld, Germany; 2 Institute of Process Engineering in Life Sciences, Department of Chemical and Process Engineering, Karlsruhe Institute of Technology, Karlsruhe, Germany

**Keywords:** carotenoids, Corynebacterium glutamicum, directed evolution, EvolvR, plasmid design

## Abstract

The EvolvR system was developed as a tool for the continuous diversification of user-defined genomic loci in *Escherichia coli*. To make EvolvR accessible for enzyme engineering in *Corynebacterium glutamicum*, this work focused on the design and construction of gpEvolvR — a modified plasmid active in both *Escherichia coli* and *Corynebacterium glutamicum* with additional recombinant features enabling its complete curing after mutagenesis. First, the pSC101 origin of replication of gpEvolvR itself served as the proof-of-principle mutagenesis target. Two examined *E. coli* mutants exhibited substantial increases in kanamycin minimal inhibitory concentrations of +44% and +96% and thus confirmed the functionality of the new gpEvolvR plasmid system. Secondly, the EvolvR-based directed evolution of a genomically located gene was shown in *C. glutamicum*. Given that the β-carotene ketolase (CrtW) has been identified as a key optimization target in previous metabolic engineering studies of *C. glutamicum*, EvolvR was successfully applied to generate mutants with varying activities, which were identified through plate-based screening. Although none of the examined *crtW* mutants outperformed the reference sequence in production experiments, this study introduced the newly constructed and curable EvolvR system as a tool for targeted hypermutation in *C. glutamicum*.

## 1 Introduction

**FIGURE 1:**
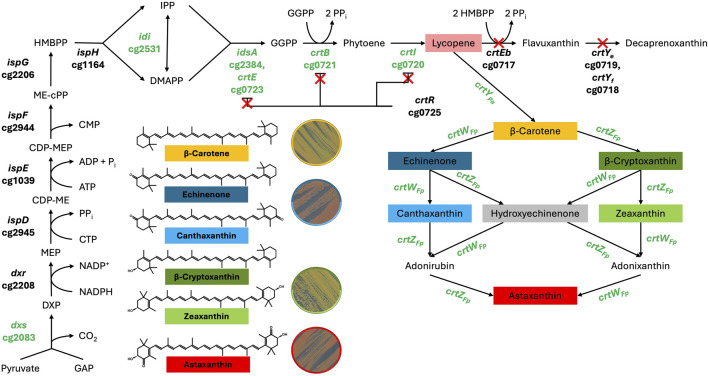
Schematic representation of astaxanthin biosynthesis in recombinant *Corynebacterium glutamicum* beginning with pyruvate and GAP. The corresponding genes encoding the catalyzing enzymes are shown next to the respective reactions. Native genes are assigned *Corynebacterium glutamicum* locus identifiers, while heterologous genes are marked with strain-specific abbreviations (Pa: *Pantoea ananatis*; Fp: *Fulvimarina pelagi*). Overexpressed genes are highlighted in green, whereas the reactions catalyzed by gene products of deleted genes are crossed out in red. Enzymes being encoded by the corresponding genes are as the following: *dxs* (cg2083): 1-deoxy-D-xylulose-5-phosphate (DXP) synthase; *dxr* (cg2208): DXP reductoisomerase; *ispD* (cg2945): 2-C-methyl-D-erythritol 4-phosphate cytidylyltransferase; *ispE* (cg1039): 4-(cytidine 5′-diphospho)-2-C-methyl-D-erythritol kinase; *ispF* (cg2944): 2-C-methyl-D-erythritol 2,4-cyclodiphosphate synthase; *ispG* (cg2206): 4-hydroxy-3-methylbut-2-enyl diphosphate synthase (HMBPP) synthase; *ispH* (cg1164): HMBPP reductase; *idi* (cg2531): isopentenyl pyrophosphate isomerase; *idsA* (cg2384)/*crtE* (cg0723): geranylgeranyl pyrophosphate synthase; *crtB* (cg0721): phytoene synthase; *crtI* (cg0720): phytoene desaturase; *crtR* (cg0725): MarR-type transcriptional regulator; *crtEb* (cg0717): lycopene elongase; *crtY*_*e*_ *Y*_*f*_ (cg0719/cg0718): C50 carotenoid ε-cyclase (heterodimeric cyclase subunits); *crtY*_*Pa*_: lycopene β-cyclase from *Pantoea ananatis*; *crtZ*_*Fp*_: β-carotene hydroxylase from *Fulvimarina pelagi*; *crtW*_*Fp*_: β-carotene ketolase from *Fulvimarina pelagi*; intermediates: DXP: 1-deoxy-D-xylulose-5-phosphate; MEP: 2-C-methyl-D-erythritol 4-phosphate; CDP-ME: 4-diphosphocytidyl-2-C-methyl-D-erythritol; CDP-MEP: 4-diphosphocytidyl-2-C-methyl-D-erythritol 2-phosphate; MEcPP: 2-C-methyl-D-erythritol 2,4-cyclodiphosphate; HMBPP: (E)-4-hydroxy-3-methylbut-2-enyl pyrophosphate; IPP: isopentenyl pyrophosphate; DMAPP: dimethylallyl pyrophosphate; GGPP: geranylgeranyl pyrophosphate. Chemical structures of synthesized carotenoids and xanthophylls are accompanied by images of corresponding producer strain colonies.

Industrial biotechnology has become a key pillar of modern production of bulk chemicals and other value-added compounds. *Corynebacterium glutamicum* is a well-established cell factory in white biotechnology, famous for its large-scale production of the amino acids L-glutamate and L-lysine (Wendisch, 2020). Moreover *C. glutamicum* is a natural producer of the carotenoid decaprenoxanthin, giving it its yellow pigmentation (Hayashi et al., 2018; Heider et al., 2012; Henke et al., 2017; Sandmann, 1994; Stegelmann et al., 2026). In response to the rising demand for carotenoids and xanthophylls within the industry (Berman et al., 2015), the natural pathway was modified to access the high-value product astaxanthin with this organism (Figure 1) (Henke et al., 2016). Platform strain engineering was conducted applying different metabolic engineering strategies in order to transform *C. glutamicum* into a producer of the central carotenoid lycopene (Heider et al., 2014; Heider et al., 2012). To expand the production portfolio towards astaxanthin, the expression of three heterologous genes was required. Branching off from lycopene, its cyclization into the orange carotenoid β-carotene was enabled by the expression of a lycopene cyclase (CrtY_Pa_) encoded by the *crtY*_*Pa*_ gene from *Pantoea ananatis* (Heider et al., 2014). Finally, astaxanthin production was established by additional expression of a β-carotene ketolase (CrtW) and a β-carotene hydroxylase (CrtZ) encoded by the respective genes *crtW*_*Fp*_ and *crtZ*_*Fp*_ from *Fulvimarina pelagi* (Henke et al., 2016). The fusion of CrtZ_Fp_ and CrtW_Fp_ (CrtZ_Fp_ ∼ CrtW_Fp_) via a flexible linker (Henke and Wendisch, 2019) balanced astaxanthin production in *C. glutamicum* (Göttl et al., 2024). However, complete β-carotene conversion could not be achieved, suggesting bottlenecks in the reactions catalyzed by β-carotene ketolase (CrtW_Fp_) and β-carotene hydroxylase (CrtZ_Fp_) as similar reported for astaxanthin biosynthesis in other microbial hosts (Lu et al., 2017; Zhang et al., 2022).

Metabolic engineering and enzyme optimization are central strategies to improve product titers and expand the product portfolio of microorganisms for useful purposes. Classical enzyme engineering strategies such as random mutagenesis do not rely on sequence-structure-function understanding. However, such approaches are limited in terms of library size, specificity and labor intensities (Bornscheuer et al., 2012; Packer and Liu, 2015). Especially the last aspect can be addressed with *in vivo* approaches, that require only initial setup for autonomous operation. Besides more traditional *in vivo* mutagenesis protocols that induce DNA damage using chemical mutagens or physical irradiation, mutator strains with defective DNA repair systems served as safer alternatives for genome-wide mutagenesis. As sequencing became broadly affordable (Shendure and Aiden, 2012), many databases and tools have contributed to a much better understanding of the sequence-structure-function relationship of encoded proteins (Kewalramani et al., 2023; Kroll et al., 2023; Pereira and Alva, 2021). Today, prediction of the impact of mutations is accelerated by artificial intelligence and combined with omics technologies, which allows for precise interventions into cellular metabolism (Fletcher et al., 2022). Over the past years the repertoire of available *in vivo* genetic engineering techniques for the precise alteration of DNA sequences has expanded significantly. Zinc Finger Nucleases (ZFN) (Kim et al., 1996) and Transcription Activator-Like Effector Nucleases (TALENs) (Boch et al., 2009) were recently surpassed by editing via clustered regularly interspaced short palindromic repeats (CRISPR). Modern CRISPR gene editing only requires cloning of a suitable protospacer upstream of a crRNA-tracrRNA fusion transcript to create an expressible sgRNA, which serves as a targeting handle for the CRISPR-associated (Cas) nuclease (Jinek et al., 2012; Mali et al., 2013). This sgRNA-nuclease complex specifically targets and cleaves double-stranded DNA at nuclease-specific protospacer adjacent motifs (PAM) (Mojica et al., 2009). Beyond double-strand nucleases for insertions and deletions, nicking nucleases (nCas) that create single-strand breaks (Cong et al., 2013) and catalytically dead versions (dCas) that retain targeting ability without cleavage (Qi et al., 2013) are established. These variants exploit the precise targeting capabilities of Cas proteins for diverse applications, including repression of gene expression (Göttl et al., 2021; Schultenkämper et al., 2019).

The EvolvR system is known as a technique combining aspects of random mutagenesis and rational design for the evolution of enzymes through diversification of user-defined loci in *E. coli* (Halperin et al., 2018). By fusing an enhanced Cas9 nickase (enCas9) (Slaymaker et al., 2016) to the N-terminus of a DNA polymerase I in which several introduced mutations led to a low-fidelity phenotype (PolI5M) (Camps et al., 2003; Loh et al., 2010), a CRISPR-guided error-prone DNA polymerase was generated for the mutagenesis of targeted regions. Thus, EvolvR can be understood as a hybrid approach combining elements of randomized mutagenesis and rational design. The concept of the EvolvR system (Halperin et al., 2018) operates as the following: (i) the DNA strand complementary to the co-expressed sgRNA (complementary strand) is nicked three base pairs upstream of the PAM, mediated by the Cas9 nuclease variant with a D10A mutation in the RuvC-like domain (enCas9) (Jinek et al., 2012), (ii) dissociation of the nickase (enCas9) from the DNA, (iii) the fused error-prone DNA polymerase (PolI5M) binds to the nick and performs mutagenic strand-displacement DNA synthesis, (iv) the displaced strand is then cleaved and degraded by the intrinsic flap endonuclease activity of the DNA polymerase, (v) the remaining nick is ligated by the endogenous DNA repair system.

Adaptive laboratory evolution (ALE) arose as a technique to mimic natural evolution in a laboratory setting by applying strong to lethal selective pressure (Elena and Lenski, 2003), e.g., forcing *C. glutamicum* cells to adapt to certain conditions such as the utilization of novel carbon sources (Werner et al., 2023) or the increasing tolerance towards toxic compounds and inhibitors (Leßmeier and Wendisch, 2015; Wang X. et al., 2018). Although EvolvR poses a powerful tool to enhance ALE processes in a continuous directed evolution (CDE) format by introducing additional variance into targeted relevant regions, it can also be applied without additional selective pressure. Together with a suitable screening method substituting for natural selection, this can allow for the directed evolution of enzymes whose products (e.g., carotenoids) could not be subjected to conventional ALE before.

However, this would require the possibility to halt the mutagenic process of EvolvR to ensure a stable relationship between genotype and phenotype for a subsequent mutant characterization via screening and gene sequencing. Although the expression of the EvolvR fusion protein from the pEvolvR-*enCas9*-*polI5M* plasmid can be induced by anhydrotetracycline (aTc), it cannot be stopped. Therefore, a proper curing process needs to be established to allow the use of the EvolvR system in such a directed evolution scenario. Furthermore, EvolvR was developed for *E. coli* and has already been adapted to yeast (Tou et al., 2020) and mammalian cells (Hurtado et al., 2025), but has not yet been transferred to the industrial workhorse *C. glutamicum*.

The aim of this study was to construct a dual-inducible and curable EvolvR-based plasmid-borne system that allows for the targeted mutagenesis in the industrial workhorses *C. glutamicum* and *E. coli*. The core value of the established system allows the rapid generation of mutant libraries in *C. glutamicum* without extensive *in vitro* cloning effort. Moreover, when combined with ALE and suitable screening assays, it can accelerate the design of enzyme properties in academic and applied research.

## 2 Materials and methods

### 2.1 General cultivation conditions of *E. coli* and *C. glutamicum*

*E. coli* and *C. glutamicum* cultures (Table 1) were cultivated in volumes of 10 mL or 50 mL in 100 mL or 500 mL baffled flasks, respectively. *E. coli* was incubated in an Ecotron shaker (Infors HT, Bottmingen, Switzerland) at 180 rpm and 37 °C. *C. glutamicum* cultures were incubated in a climate chamber at 120 rpm (corresponding to 0.402 × g) and 30 °C. Where applicable, cultures containing plasmids were supplemented with vector-specific antibiotics tetracycline (5 μg mL^−1^), kanamycin (25 μg mL^−1^), chloramphenicol (7.5 μg mL^−1^) (VWR International GmbH, Darmstadt, Germany) to maintain selective pressure.

**TABLE 1:** Plasmids used in this study.

Plasmid	Features	References
pECXT-P_Syn_-*crtW*_*Fp*_	Tet^R^, pEC-XT99A-P_Syn_ derivative for constitutive expression of *crtW* from *F. pelagi* under the control of the synthetic P_Syn_ promoter (Rytter et al., 2014)	Göttl et al. (2024)
pECXT-P_tuf_-*crtW*_*Fp*_	Tet^R^, pEC-XT99A-P_tuf_ derivative for constitutive expression of *crtW* from *F. pelagi* under the control of the P_tuf_ promoter from *C. glutamicum*	This work
pK19*mobsacB*	pK18 (oriV_Ec_, *sacB, lacZ*_*α*_) Km^R^, *C. glutamicum/E. coli* expression shuttle vector for construction of genomic insertion and deletion mutants of *C. glutamicum*	Schäfer et al. (1994)
pK19*mobsacB*Δ*actA*::P_H36_-*crtW*_*Fp*_	Km^R^, pK19*mobsacB* derivative for the deletion of the *actA* locus and integration of the *crtW* gene from *F. pelagi* under the control of the P_H36_ promoter (Yim et al., 2013)	This work
pK19*mobsacB*Δ*actA*::P_tuf_-*crtW*_*Fp*_	Km^R^, pK19*mobsacB* derivative for the deletion of the *actA *locus and integration of the *crtW* gene from *F. pelagi* under the control of the P_tuf_ promoter from *C. glutamicum*	This work
pK19*mobsacB*Δ*actA*::P_Syn_-*crtW*_*Fp*_	Km^R^, pK19*mobsacB* derivative for the deletion of the *actA *locus and integration of the *crtW* gene from *F. pelagi* under the control of the P_Syn_ promoter	This work
pEvolvR-*enCas9*-*polI5M*	Km^R^, oriV_Ec_, contains translational fused enCas9 nickase and PolI5M (*E. coli* DNA polymerase I harboring D424A, I709N, A759R, F742Y and P796H mutations) followed by the synthetic L3S2P55 terminator, sgRNA expression cassette	Halperin et al. (2018), Addgene Plasmid #113078
pSH1	Km^R^, P_tuf_, pHM519 oriV_Cg_ *, C. glutamicum/E. coli* expression shuttle vector	Henke et al. (2016)
pSH1-EvolvR	Km^R^, pSH1 derivative containing *Sal*I and *Pst*I cured *enCas9*-*polI5M* sequence	This work
pJYS3_easy cloning	Km^R^, pJYS3_Δ*crtY*_*ef*_ (Jiang et al., 2017) derivative; Km^R^, repA101, pSC101 ori *E. coli* replicon, pBL1_ts_ temperature sensitive *C. glutamicum* replicon, cpf1 nuclease from *Francisella novicida* under the control of the constitutive P_lacM_ promoter, rrnB T_2_ Terminator	This work
pS_dCas9	Cm^R^, pRG_dCas9 (Gauttam et al., 2019) derivative carrying the Cas9 handle followed by the terminator from *Streptococcus pyogenes* amplified from the piCas plasmid (Schultenkämper et al., 2019)	Göttl et al. (2021)
gpEvolvR	Km^R^, repA101, pSC101 ori *E. coli* replicon, pBL1_ts_ temperature sensitive *C. glutamicum* replicon, *tetO/tetR* regulated expression of *Sal*I and *Pst*I cured *enCas9-polI5M* sequence, *lacI* ^*q*^ inducible sgRNA scaffold	This work
gpEvolvR -HIS_1_	Km^R^, gpEvolvR derivative containing a 20bp protospacer targeting the area upstream of the first conserved histidine rich region within the *crtW* gene from *F. pelagi*	This work
gpEvolvR -HIS_1-2_	Km^R^, gpEvolvR derivative containing a 20bp protospacer targeting the area upstream between the first and second conserved histidine rich region within the *crtW* gene from *F. pelagi*	This work
gpEvolvR -HIS_3_	Km^R^, gpEvolvR derivative containing a 20bp protospacer targeting the area upstream of the third conserved histidine rich region within the *crtW* gene from *F. pelagi*	This work
pSH1-*crtW*_Fp_*MX	Km^R^, pSH1 derivative containing a *crtW*_*Fp*_ variant	This work
gpEvolvR-ORI_2_	Km^R^, gpEvolvR derivative containing a 20bp protospacer targeting the pSC101 ori within gpEvolvR	This work
gpEvolvR-ORI_2_^X1^	Km^R^, gpEvolvR derivative containing a 20bp protospacer targeting the pSC101 ori within gpEvolvR harboring a T318A SNP in the pSC101 ori	This work
gpEvolvR-ORI_2_^X9^	Km^R^, gpEvolvR derivative containing a 20bp protospacer targeting the pSC101 ori within gpEvolvR harboring a T395C SNP in the pSC101 ori	This work

### 2.2 General cloning strategy

Molecular cloning was performed via Gibson assembly (Gibson et al., 2009). In brief, target DNA fragments were amplified with a High-Fidelity DNA Polymerase (Allin™, highQu GmbH, Kraichtal, Germany). To enhance amplification efficiency dimethyl sulfoxide (DMSO) and betaine were added to the reaction mix. Plasmids were isolated from *E. coli* DH5α 10 mL or 50 mL overnight LB cultures (plus appropriate antibiotics) using the QIAwave Plasmid Miniprep Kit (QIAGEN GmbH, Hilden, Germany) according to the manufacturer’s protocol. Plasmids were linearized with the respective restriction enzyme (Thermo Fisher Scientific Baltics UAB, Vilnius, Lithuania) and subsequently dephosphorylated with Antarctic Phosphatase (New England Biolabs GmbH, Frankfurt, Germany). PCR products and restricted plasmids were purified either from suspension or agarose-gels by using the NucleoSpin™ Gel and PCR Clean-Up Kit (Macherey-Nagel GmbH & Co. KG, Düren, Germany). Linearized plasmids and insert DNA were used for the Gibson assembly (Gibson et al., 2009). Chemically competent *E. coli* DH5α cells were used for subcloning of all new plasmid constructs (Table 2), that were subsequently tested by colony PCR (cPCR). Sequencing of DNA was either performed as Sanger sequencing at the Sequencing Core Facility in the Center for Biotechnology at Bielefeld University (Bielefeld, Germany) or as Oxford Nanopore Technologies (ONT) sequencing externally at Microsynth Seqlab GmbH (Göttingen, Germany).

**TABLE 2:** Strains used in this study.

Strain	Features	References
*E. coli* strains		
*E. coli* DH5α	F^−^ endA1 hsdR17 (r^-^_k_,m^+^_k_) supE44 thi-1 λ^−^ recA1 gyrA96 relA1 deoR Δ(lacZYA-argF)-U169φ 80dlacZ*Δ*M15	Hanahan (1983)
*E. coli* DH5α (gpEvolvR-ORI_2_)	*E. coli* DH5α derivative containing the gpEvolvR-ORI_2_ plasmid	This work
*E. coli* DH5α (gpEvolvR-ORI_2_^X1^)	*E. coli* DH5α derivative containing the gpEvolvR-ORI_2_^X1^ plasmid	This work
*E. coli* DH5α (gpEvolvR-ORI_2_^X9^)	*E. coli* DH5α derivative containing the gpEvolvR-ORI_2_^X9^ plasmid	This work
*E. coli* DH5α (pVWEx1)	*E. coli* DH5α derivative containing the pVWEx1 plasmid	Peters-Wendisch et al. (2001)
*C. glutamicum* strains		
*C. glutamicum* BETA4	*C. glutamicum* MB001 derivative producing β-carotene	Henke et al. (2016)
*C. glutamicum* BETA4 (pSH1-*crtW*_*Pn*_)	*C. glutamicum* BETA4 derivative containing the pSH1-*crtW*_*Pn*_ plasmid for constitutive expression of codon optimized *crtW* encoding for CrtW from *Paracoccus* N81106 with M99I/L175M mutations	This work
*C. glutamicum* BETA4 (pSH1-*crtW*_*Sd*_)	*C. glutamicum* BETA4 derivative containing the pSH1-*crtW*_*Pn*_ plasmid for constitutive expression of codon optimized *crtW* encoding for CrtW from *Sphingomonas *DC18 with R203W/F213L mutations	This work
*C. glutamicum* BETA4 (pSH1-*crtW*_*Fp*_)	*C. glutamicum* BETA4 derivative containing the pSH1-*crtW*_*Fp*_ plasmid for constitutive expression of *crtW* from *Fulvimarina pelagi*	Henke et al. (2016)
*C. glutamicum* BETA4 Δ*actA*::P_H36_-*crtW*_*Fp*_	*C. glutamicum* BETA4 derivative with integration of the *crtW*_*Fp*_ into the *actA* (cg2840) locus of the *C. glutamicum* genome for constitutive expression under control of the P_H36_ promoter	This work
*C. glutamicum* BETA4 Δ*actA*::P_tuf_-*crtW*_*Fp*_	*C. glutamicum* BETA4 derivative with integration of the *crtW*_*Fp*_ into the *actA* (cg2840) locus of the *C. glutamicum* genome for constitutive expression under control of the P_tuf_ promoter	This work
*C. glutamicum* BETA4 Δ*actA*::P_Syn_-*crtW*_*Fp*_	*C. glutamicum* BETA4 derivative with integration of the *crtW*_*Fp*_ into the *actA* (cg2840) locus of the *C. glutamicum* genome for constitutive expression under control of the P_Syn_ promoter	This work
*C. glutamicum* BETA4 Δ*actA*::P_Syn_-*crtW*_*Fp*_ (gpEvolvR-HIS_1_)	*C. glutamicum* BETA4 derivative with P_Syn_-*crtW*_*Fp*_ integrated into the *actA* (cg2840) locus of the *C. glutamicum* genome harboring the gpEvolvR-HIS_1_ plasmid for mutagenesis of *crtW*_*Fp*_	This work
*C. glutamicum* BETA4 Δ*actA*::P_Syn_-*crtW*_*Fp*_ (gpEvolvR-HIS_1-2_)	*C. glutamicum* BETA4 derivative with P_Syn_-*crtW*_*Fp*_ integrated into the *actA* (cg2840) locus of the *C. glutamicum* genome harboring the gpEvolvR-HIS_1-2_ plasmid for mutagenesis of *crtW*_*Fp*_	This work
*C. glutamicum* BETA4 Δ*actA*::P_Syn_-*crtW*_*Fp*_ (gpEvolvR-HIS_3_)	*C. glutamicum* BETA4 derivative with P_Syn_-*crtW*_*Fp*_ integrated into the *actA* (cg2840) locus of the *C. glutamicum* genome harboring the gpEvolvR-HIS_3_ plasmid for mutagenesis of *crtW*_*Fp*_	This work
*C. glutamicum* BETA4 (pSH1-*crtW*_Fp_*MX)	*C. glutamicum* BETA4 derivative containing a pSH1-*crtW*_Fp_*MX plasmid	This work
*C. glutamicum* BETA4 (pECXT-P_Syn_-*crtZ*_*Fp*_)	*C. glutamicum* BETA4 derivative containing a pECXT-P_Syn_-*crtZ*_*Fp*_ plasmid for constitutive expression of the *crtZ* gene from *F. pelagi*	Meyer et al. (2025)
*C. glutamicum* BETA4 (pECXT-P_Syn_-*crtZ*_*Fp*_) (pSH1-*crtW*_*Fp*_)	*C. glutamicum* BETA4 derivative containing both plasmids pECXT-P_Syn_-*crtZ*_*Fp*_ and pSH1-*crtW*_*Fp*_ for constitutive expression of both genes *crtZ* and *crtW* from *F. pelagi*	This work
*C. glutamicum* BETA4 (pSH1-*crtW*_Fp_*MX) (pECXT-P_Syn_-*crtZ*_*Fp*_)	*C. glutamicum* BETA4 derivative containing both plasmids pSH1-*crtW*_Fp_*MX and pECXT-P_Syn_-*crtZ*_*Fp*_	This work

### 2.3 Construction of the gpEvolvR plasmid

As protospacer integration was aimed for at *Pst*I or *Sal*I sites in the new construct, undesired restriction motifs had to be removed from affected sequences. For this purpose, a 7,754 bp fragment of the plasmid pEvolvR-*enCas9*-*polI5M* (Halperin et al., 2018) (Addgene Plasmid #113078), comprising *tetR*, *enCas9-polI5M*, and other regulatory elements, was divided into three fragments. The first fragment, containing *tetR* and part of the *enCas9* sequence, was synthesized as a 1156 bp gene fragment (Twist Bioscience, San Francisco, United States) with both internal *Sal*I sites removed. The synthesized fragment was then flanked with matching overhangs by high-fidelity PCR using primers 037P and 038P. The second (5507 bp) and third (1091 bp) fragment were amplified via high-fidelity PCR using primers 039P/040P and 041P/042P, respectively, with primers 040P and 041P introducing the necessary mismatches for the removal of an internal *Pst*I site by single nucleotide substitution. All three fragments were stored by assembly into the pSH1 backbone yielding the construct pSH1-EvolvR. For construction of gpEvolvR, the insert of pSH1-EvolvR (043P/044P) as well as the *lacI* ^*q*^ regulated sgRNA scaffold from pS_dCas9 (045P/046P) and *kanR*, pBL1_ts_, pSC101 ori and *repA101* from pJYS3_easy cloning (047P/048P) were amplified and assembled.

### 2.4 Protospacer integration into the gpEvolvR system

Suitable protospacers were identified employing *CRISPy-web* (crispy.secondarymetabolites.org) (Blin et al., 2016) with the corresponding host genome as reference to minimize potential off-target effects. Protospacer inserts were generated by the annealing of two complementary oligonucleotides (Sigma-Aldrich Chemie GmbH, Taufkirchen, Deutschland) (ORI_2_: 068P/069P; HIS_1_: 083P/084P; HIS_1-2_: 085P/086P; HIS_3_: 089P/090P). 5 μL of each equimolar oligonucleotide solution (100 µM) was added to 990 µL of annealing buffer. The resulting mixture was heated to 95 °C for 5 min and then gradually cooled to room temperature. Subsequently annealed oligonucleotides were assembled into the *Pst*I-restricted and dephosphorylated gpEvolvR backbone via Gibson assembly (Gibson et al., 2009). Successful protospacer integration was verified via cPCR and confirmed by Sanger sequencing using the primers vgai/076P.

### 2.5 *In vivo* mutagenesis of the pSC101 ori by gpEvolvR in *E. coli*

A 10 mL LB_Km25_ overnight culture of *E. coli* DH5α (gpEvolvR-ORI_2_), inoculated from a glycerol stock, was diluted to an OD_600nm_ of 0.02 using sterile LB medium. Subsequently, 100 µL of the diluted cell suspension were plated onto LB agar plates containing 500 μg mL^−1^ kanamycin, 0.25 μg mL^−1^ anhydrotetracycline (aTc), and 1 mM isopropyl-β-D-thiogalactopyranoside (IPTG). Plates were incubated at 37 °C for approximately 24 h. After incubation, individual colonies were transferred to LB_Km1000_ agar plates to isolate clones, apply higher selective pressure, and remove the inducers to stop the mutagenic process.

The targeted pSC101 ori (029P/097P) and the kanamycin resistance cassette (*kanR*) including 250 bp upstream regulatory elements (035P/091P) were amplified via cPCR. Amplicons were treated with ExoSAP-IT™ (Applied Biosystems™, Thermo Fisher Scientific, Waltham, USA) and subjected to Sanger sequencing using primers 029P and 097P for the pSC101 ori, and 035P, 036P, 091P, and 100P for *kanR*. Clones containing unique *de novo* mutations in the pSC101 ori but no mutations within the amplified *kanR* region were selected and preserved as glycerol stocks for further experiments.

Overnight precultures of *E. coli* strains were harvested by centrifugation at 4,000 × g for 7 min and washed twice with TN buffer. The cell pellet was resuspended in 1 mL LB medium to an OD_600nm_ of 1.0 using a V-1200 spectrophotometer (VWR International bvba, Leuven, Belgium). For each well of a flat-bottom 24-well microtiter plate equipped with a CR1224b sandwich cover (System Duetz, EnzyScreen BV, Heemstede, Netherlands), 990 µL of LB medium containing the appropriate antibiotics were mixed with 110 µL of the prepared cell suspension. To determine the initial cell density (t_0_), 100 µL of culture were withdrawn from each well for OD_600nm_ measurement, leaving 1 mL in each well. Cultivation was carried out in an Ecotron shaker (Infors HT, Bottmingen, Switzerland) at 180 rpm and 37 °C for 24 h.

### 2.6 *In vivo* mutagenesis of *crtW*_*Fp*_ by gpEvolvR in *C. glutamicum*

A 10 mL CGXII_opt/Km25_ culture of *C. glutamicum* BETA4 Δ*actA*::P_Syn_-*crtW*_*Fp*_ cells containing 60 mM acetate and 20 mM glucose as carbon sources was inoculated from LB_Km25_-agar plates. After 24 h of incubation, expression of the EvolvR fusion protein and the sgRNA was induced by supplementation of 0.25 μg mL^−1^ aTc and 1 mM IPTG respectively and cultivation continued for another 24 h. Cells were diluted to an OD_600nm_ of 1 × 10^−5^ and 100 µL of the cell suspension was plated onto CGXII_opt/Km25_ agar plates (Göttl et al., 2024) containing 60 mM acetate and 20 mM glucose as carbon sources.

Plates were incubated at 37 °C for plasmid curing via the pBL1_ts_ origin of replication (Nakamura et al., 2006) and an optical screening was performed after 2–4 days of incubation. Selected clones were transferred to both a CGXII_opt_ agar plate without antibiotics and one with 25 μg mL^−1^ kanamycin to verify the loss of the gpEvolvR plasmid. Clones sensitive to kanamycin were optically re-screened after 24–72 h of incubation and high-fidelity cPCR using the primers 051P/052P for *crtW*_*Fp*_ and V680/V099 for *crtY*_*Pa*_ respectively was performed on selected clones. Prepared amplicons were subjected to Sanger sequencing to detect possible mutations.

### 2.7 Chromosomal mutant construction of *C. glutamicum* genome via homologous recombination using pK19*mobsacB*

For the integration of genes into the *actA* (cg2840) locus of the *C. glutamicum* genome, both 1 kb upstream and downstream flanking regions of the *actA* locus were amplified via PCR from genomic DNA, while inserts were amplified from plasmids pECXT-P_Syn_-*crtW*_*Fp*_, pECXT-P_tuf_ *-crtW*_*Fp*_. In case of P_H36_, the promoter was synthetically introduced via oligoextension with the primer pair 058P/059P. Primers with specific overhangs were used to generate compatible ends between the pK19*mobsacB* backbone, the flanking regions, and the inserts, respectively.

A Gibson assembly (Gibson et al., 2009) was performed using the prepared *Bam*HI-restricted and dephosphorylated pK19*mobsacB* backbone. The assembled construct was transformed into chemically competent *E. coli* DH5α cells for plasmid amplification. After verification of plasmid uptake via cPCR, plasmid DNA was isolated, and relevant regions were sequenced to confirm correct assembly. Confirmed plasmids were electroporated into electrocompetent *C. glutamicum* cells, and transformants were selected on BHIS (kanamycin 25 μg mL^−1^) agar at 30 °C for 2 days. Clones were subsequently screened on LB (kanamycin 25 μg mL^−1^ and sucrose 100 g L^−1^) and LB (kanamycin 25 μg mL^−1^) plates to identify kanamycin-resistant, sucrose-sensitive colonies, which were further streaked on LB (sucrose 100 g L^−1^) to select for the second recombination event. Kanamycin-sensitive, sucrose-resistant clones were finally verified by cPCR using primers 055P and 056P and the correct genomic integrations were confirmed by sequencing with primers 063P, V201, V209 and 1023.

### 2.8 Production experiments with *crtW*_*Fp*_ mutant variants

To assess *crtW*_*Fp*_ mutants under production conditions, the *crtW*_*Fp*_ gene was amplified from selected mutant strains via high-fidelity PCR using the primers 103P and 102P, adding overhangs for integration into *Bam*HI restricted pSH1 backbone via Gibson assembly (Gibson et al., 2009). Assembled constructs were transformed into chemically competent *E. coli* DH5α cells and plasmid uptake was verified by cPCR using the primers 1129 and 1135. The plasmids of positive clones were isolated and Sanger sequencing confirmed the sequence of mutant *crtW*_*Fp*_.

To compare the activity of mutant *crtW*_*Fp*_ alone and in combination with *crtZ*_*Fp*_, the plasmids were electroporated into both *C. glutamicum* BETA4 and *C. glutamicum* BETA4 (pECXT-P_Syn_-*crtZ*_*Fp*_) cells. For verification of correct transformation, high-fidelity cPCR was performed on transformants using the primers 1129/1135 and Sanger sequencing was performed on prepared amplicons using the primers 1129/1135/V201/V209.

Precultures of cloned mutant strains and corresponding reference strains were grown in 10 mL LB medium supplemented with 10 g L^−1^ glucose overnight. Technical triplicates of main cultures were grown in 10 mL CGXII_opt_ minimal medium (Göttl et al., 2024) supplemented with 40 g L^−1^ of glucose and appropriate antibiotics after washing twice in 10 mL sterile TN-buffer and resuspending the cells in prepared minimal medium for inoculation to an initial OD_600nm_ of 1. After 72 h of cultivation, the optical densities were measured using a Spectrophotometer V-1200 (VWR International, Leuven, Belgium) and 500 µL of the cell suspension were centrifuged at 20,238 × g and the pellet was frozen at −20 °C until extraction and analysis.

### 2.9 Extraction and quantification of carotenoids

The extraction of carotenoids was carried out by resuspension of the cell pellet in 1000 µL of a methanol:acetone (7:3; [v/v]) mixture containing butylated hydroxytoluene (BHT) as an antioxidant. Resuspended cells were shaken for 30 min at 1000 rpm and 60 °C using a ThermoMixer® C (Eppendorf SE, Hamburg, Germany), followed by centrifugation at 20,238 × g for at least 10 min. The supernatant was transferred to glass vials, and the carotenoid content was quantified via high-performance liquid chromatography (HPLC) according to the protocol described by (Göttl et al., 2023).

## 3 Results

### 3.1 Design and construction of gpEvolvR

**FIGURE 2:**
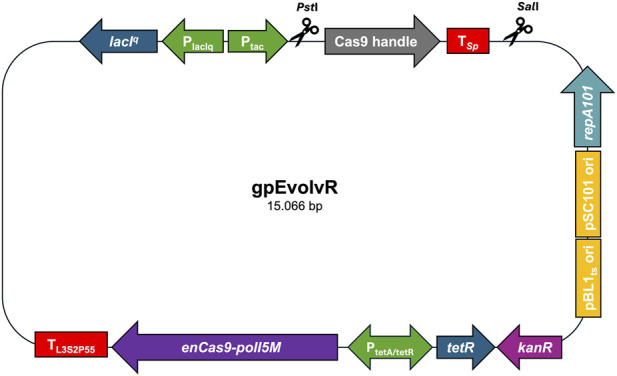
Schematic organization of features on the constructed gpEvolvR plasmid. The size of the features and the distances between them are not shown to scale. Operators associated with P_*tac*_ and P_*tetA*_, which are located downstream of the promoter sequences in close proximity, are not shown. A list of the incorporated features and their functions can be found in Supplementary Table S2.

The new plasmid gpEvolvR (Figure 2) was constructed by recombining features from pEvolvR-*enCas9*-*polI5M*, pJYS3_easy cloning, and pS_dCas9 (Supplementary Table S2). The combination of these features enables a targeted mutagenesis approach based on a plasmid replicating in both *E. coli* and *C. glutamicum* as hosts that offers aTc inducible expression of *enCas9*-*polI5M* (Halperin et al., 2018) and IPTG-inducible Cas9 handle expression and that is curable via temperature sensitive origin of replication pBL1_ts_ (Nakamura et al., 2006). Protospacer DNAs can be integrated via *Pst*I restriction followed by Gibson assembly (Gibson et al., 2009), and the system is prepared for multiplex mutagenesis through integration of multiple sgRNA expression cassettes as described by (Sakuma et al., 2014) (Figure 2).

### 3.2 *In vivo* mutagenesis of the pSC101 ori by gpEvolvR

**FIGURE 3:**
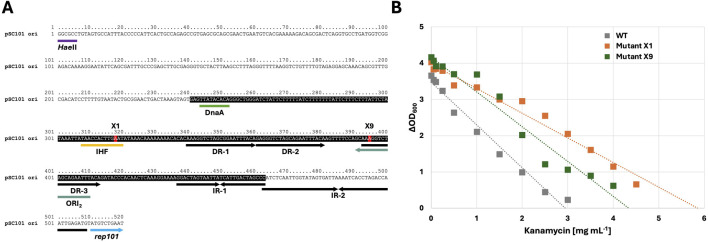
**(A)** Protospacer and obtained mutations X1 and X9 within the nucleotide sequence of the pSC101 ori. Shown sequence starts from the *Hae*II site (purple) upstream of the essential sequence reported by Sugiura et al. (1993), which is highlighted by a black background. Positions of nucleotides substituted in the mutants X1 (A → T at 318 bp) and X9 (A → G at 395 bp) are highlighted by a red background. The binding sequence of the employed protospacer ORI_2_ (teal) is displayed as an arrow indicating the direction of mutagenesis. Additionally, relevant sequences such as DnaA (green) and IHF (orange) binding sites, the first 10 base pairs of the downstream located repA101 gene sequence (blue) as well as direct repeats (DR-1, DR-2, and DR-3) and inverted repeats (IR-1 and IR-2) are marked as described by (Sugiura et al., 1993). **(B)** Impact of mutations within the pSC101 ori on growth in the presence of kanamycin. Cultivation was performed in System Duetz microtiter plates at 37 °C and 180 rpm for 24 h using LB medium supplemented with increasing kanamycin concentrations. Shown are the calculated ΔOD_600nm_ values for the *E. coli* DH5α strains carrying either the wild-type plasmid gpEvolvR-ORI_2_ (WT, grey) or the mutated plasmids gpEvolvR-ORI_2_^X1^ (X1, orange) and gpEvolvR-ORI_2_^X9^ (X9, green). Linear regressions were determined (WT, grey: f(x) = −1.2x + 3.5; Mutant X1, orange: f(x) = −0.7x + 4.0; Mutant X9, green: f(x) = −1x + 4.2) and used for MIC calculation.

EvolvR was developed for application in *E. coli*, with replication of the pEvolvR-*enCas9*-*polI5M* plasmid being initiated by the oriV origin of replication derived from plasmid pBR322 (Halperin et al., 2018). To show that the recombined plasmid gpEvolvR is still stable and EvolvR active in its original host after the exchange of the origin of replication to the pSC101 ori donated by pJYS3_easy cloning (Supplementary Table S2), a mutagenesis experiment was performed in *E. coli* DH5α cells with the pSC101 ori on gpEvolvR as the target. A corresponding protospacer (ORI_2_; Supplementary Table S1), targeting a directed repeat sequence (DR-3) of the pSC101 ori (Figure 3), was integrated upstream of the sgRNA scaffold of gpEvolvR.

After mutagenesis in *E. coli* DH5α using the gpEvolvR-ORI_2_ construct, 11 colonies were isolated from LB agar plates containing 1 mg mL^−1^ kanamycin and subjected to sequencing analysis. The gpEvolvR-ORI_2_^X1^ variant (short: X1) resulted from an A → T base substitution located 318 bp downstream of the *Hae*II site within the binding sequence of the integration host factor (IHF) (Figure 3). Nine of the analyzed clones carried the X1 variant of pSC101 ori. The second identified variant gpEvolvR-ORI_2_^X9^ (short: X9) resulted from an A → G base substitution located 395 bp downstream of the *Hae*II site within the sequence of the third direct repeat (DR-3) within the pSC101 ori (Figure 3A). Moreover, one isolated clone was able to grow under the applied selective conditions, but did not harbor a mutation within the *ori* region.

Since mutations in the pSC101 ori can be expected to influence plasmid copy number and consequently affect host resistance levels through gene dosage effects in this case of the kanamycin resistance (Potekhin and Danilenko, 1985), growth experiments were carried out using increasing concentrations of kanamycin. Since a previous E-Test on LB agar plates was not sufficient to observe any inhibitory effects by kanamycin on the strains carrying the gpEvolvR-ORI_2_ variants (Supplementary Figure S1), an experiment using liquid medium in System Duetz microtiter plates was performed.

The examined mutants X1 and X9 showed an increased resistance towards kanamycin (Figure 3B). Notably, the MIC values of mutant X9 (4.2 mg mL^−1^) and mutant X1 (5.7 mg mL^−1^) were approximately 44% and 96% higher as in the control strain (MIC: 2.9 mg mL^−1^).

### 3.3 Establishment of a CrtW screening method for EvolvR experiments

**FIGURE 4:**
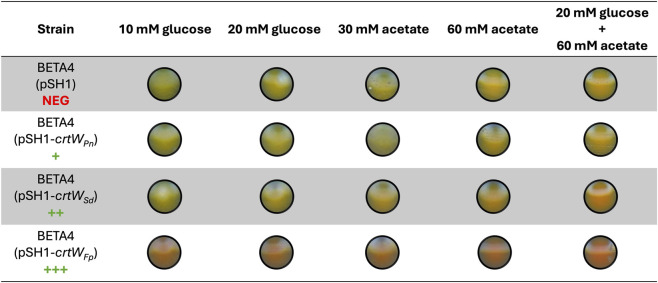
Pigmentation of single colonies from *Corynebacterium glutamicum* BETA4 strains expressing *crtW* variants on CGXII_opt/Km25_ agar plates containing different carbon source compositions. As carbon sources glucose (10 mM and 20 mM) as well as its carbon-equivalent concentrations of acetate (30 mM and 60 mM) and a combination of both (20 mM glucose and 60 mM acetate) were used. The corresponding empty vector strain *Corynebacterium glutamicum* BETA4 (pSH1) served as a reference (NEG) to the strains expressing *crtW* variants which are sorted for their activity in *Corynebacterium glutamicum* (+, ++, and +++) as reported by (Göttl et al., 2023). Plates were incubated at 30 °C for 2 days followed by incubation at room temperature for another 4 days.

In astaxanthin biosynthesis, the β-carotene ketolase (see Supplementary Figure S2) is oftentimes considered as a bottleneck (Li et al., 2020; Tao et al., 2006; Ye et al., 2006) and was thus chosen for EvolvR experiments. First, screening conditions had to be established. The ketolization of orange-colored β-carotene into the red-colored pigments echinenone and canthaxanthin produces easily detectable color changes for visual screening (Figure 1). The use of CGXII_opt_ agar medium was chosen to mimic production conditions and to reduce background pigmentation, as CGXII_opt_ itself does not exhibit any coloration similar to the relevant carotenoids β-carotene, echinenone, or canthaxanthin (Figure 1). First the optimal supplementation of carbon sources for the production of these relevant carotenoids on CGXII_opt_ agar plates needed to be determined. Therefore *C. glutamicum* BETA4 strains expressing heterologous *crtW* variants were cultivated on CGXII_opt/Km25_ agar plates supplemented with different carbon source compositions (Figure 4; Supplementary Figure S3). The resulting colony pigmentation was used as an indirect measure of carotenoid production. The tested recombinant codon optimized variants *crtW*_*PN*_ (from *Paracoccus* N81106), *crtW*_*SD*_ (from *Sphingomonas* DC18) and *crtW*_*Fp*_ (from *Fulvimarina pelagi*) in ascending order exhibit different β-carotene ketolase production profiles in *C. glutamicum* BETA4 (data not shown) allowing the optical assessment of characteristic pigment ratios.

The single colonies of the assessed strains showed variations in pigmentation as expected (Figure 4). While the empty vector strain and the *crtW*_*PN*_ variant exhibited only a yellow to bright orange coloration, increasing levels of red pigmentation could be observed for the *crtW*_*SD*_ and the *crtW*_*Fp*_ variants respectively. Also, noticeable differences in pigmentation occurred, if the composition of the provided carbon source was altered, with increasing amounts of carbon source also causing an increase in pigmentation. Furthermore, carbon-equivalent concentrations of acetate appeared to be superior regarding the promotion of pigmentation (Supplementary Figure S3; Figure 4). The most vivid coloration of colonies for all tested strains was achieved by the combined supplementation of 20 mM glucose and 60 mM acetate as carbon sources to CGXII_opt_ agar plates, hence this combination was used for all further screening experiments.

**FIGURE 5:**
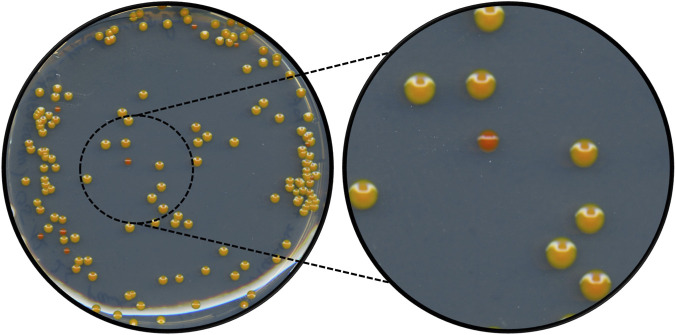
Mixture of single colonies from *Corynebacterium glutamicum* BETA4 strains expressing heterologous *crtW* variants. Cells were cultivated on CGXII_opt/Km25_ agar plates supplemented with 20 mM glucose and 60 mM acetate for 2 days at 30 °C followed by another 4 days at room temperature. The strains were mixed at a defined ratio of 40% pSH1 empty vector strain, 30% *crtW*_*PN*_, 20% *crtW*_*SD*_ and 10% *crtW*_*Fp*_ respectively.

To test if single colonies within a heterogenous group could be visually distinguished from each other as necessary for a successful screening, a mixture of the strains expressing the *crtW* variants and their respective empty vector reference strain were cultivated on CGXII_opt/Km25_ agar plates supplemented with 20 mM glucose and 60 mM acetate (Figure 5).

The colonies of the *C. glutamicum* BETA4 strain expressing *crtW*_*Fp*_ could be isolated easily due to their prominent red pigmentation (Figure 5), whereas the differentiation between the other variants proved to be difficult. Some colonies showed a rather intense orange coloring similar to the pigmentation of the *crtW*_*SD*_ variant, whereas the *crtW*_*PN*_ variant and the empty vector reference remained indistinguishable as in Figure 4.

### 3.4 Construction of target strains for *crtW*_*Fp*_ mutagenesis

**FIGURE 6:**
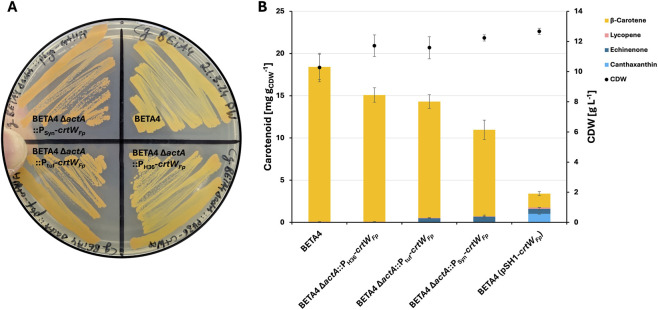
**(A)** Pigmentation of *Corynebacterium glutamicum* BETA4 Δ*actA*::*crtW*_*Fp*_ integration strains with *crtW*_*Fp*_ under the control of promoters of different strengths. Shown are the base strain BETA4 (top right) as well as the integration strains with P_H36_ promoter (bottom right), P_tuf_ promoter (bottom left) and P_Syn_ promoter (top left). Strains were streaked from glycerol stocks onto an LB agar plate and incubated at 30 °C for 48 h. **(B)** HPLC analysis and biomass concentration of *C. glutamicum* BETA4 Δ*actA*::*crtW*_*Fp*_ integration strains with *crtW*_*Fp*_ under the control of promoters of different strengths. Shown are the means of cell dry weight (CDW) and detected carotenoids relative to the CDW for 10 mL technical triplicates grown in CGXII_opt_ minimal medium supplemented with 40 g L^−1^ glucose (and kanamycin for pSH1 harboring strains) for 48 h with the respective standard deviation as Y-error.

As a direct relationship between genotype and phenotype is necessary to isolate single mutant *crtW*_*Fp*_ sequence variants after mutagenesis, a respective target strain with a genomic integration of the *crtW*_*Fp*_ gene resulting in a single copy of the target gene per cell had to be constructed. To assess different expression levels of *crtW*_*Fp*_ regarding their suitability for screenings, three integration strains with individual promotors of different expression strengths were constructed by pK19*mobsacB* mediated homologous recombination. The three selected promotors were already examined for episomal expression in *C. glutamicum* (Henke et al., 2021) and range from low (P_H36_) (Yim et al., 2013) over medium (P_tuf_) (Becker et al., 2005) to high (P_Syn_) (Rytter et al., 2014) levels of expression. As expected, the intensity of red pigmentation caused by CrtW_Fp_ products increases with promoter strength (Figure 6A).

The HPLC results of a production experiment (Figure 6B) confirmed the correlation between CrtW_Fp_ products and pigmentation, with echinenone being the main pigment produced by the integration strains. The amount of echinenone produced was similar when comparing episomal expression and genomic integrations of *crtW*_*Fp*_ under the control of the P_tuf_ or P_Syn_ promoter. However, remarkable amounts of canthaxanthin were only produced by episomal expression (Supplementary Table S3). Although the episomal expression strain BETA4 (pSH1-*crtW*_*Fp*_) is superior in terms of CrtW_Fp_ product formation, its total amount of carotenoids is dramatically reduced compared to the other strains. In fact, the data suggests a noticeable correlation between the formation of CrtW_Fp_ products and total carotenoid content (*R* ^2^ = 0.946), as there is a trend of β-carotene content being more reduced than one would expect solely from its metabolization towards echinenone and canthaxanthin (Figure 6B). For example, the strain BETA4 (pSH1-*crtW*_*Fp*_) showed an increase of 0.93 mg g_CDW_^−1^ in CrtW_Fp_ products, but a decrease of 7.54 mg g_CDW_^−1^ in its overall carotenoid content, with β-carotene being the major compound affected (Supplementary Table S3).

Since the strain BETA4 Δ*actA*::P_Syn_-*crtW*_*Fp*_ expressing *crtW*_*Fp*_ via the P_Syn_ promoter can be easily distinguished from the parental strain BETA4 or later dysfunctional *crtW*_*Fp*_ mutants on plates (Figure 6A) and leaves room for mutagenic improvement compared to the pigmentation and carotenoid content observed with episomal expression (Figure 6), this variant was chosen as the target strain for the mutagenic experiments.

### 3.5 *In vivo* mutagenesis of *crtW*_*Fp*_ by gpEvolvR

**FIGURE 7:**
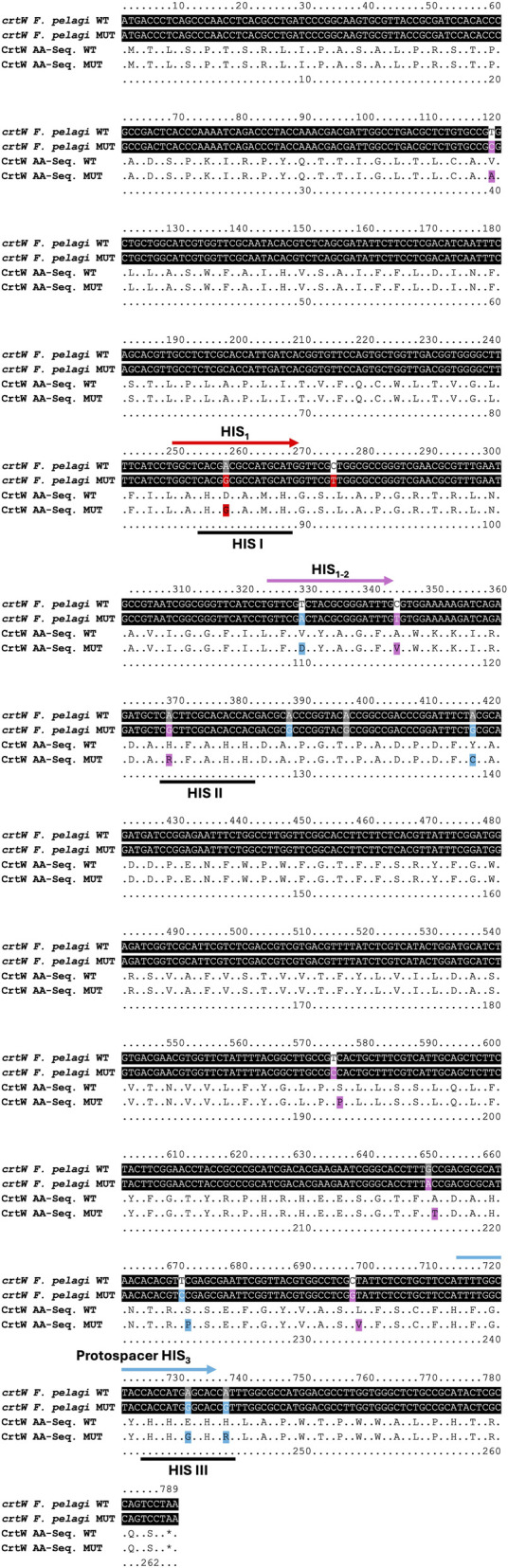
Positions of identified single nucleotide polymorphisms (SNPs) in *crtW*_*Fp*_ relative to the protospacer sequences employed for mutagenesis with gpEvolvR. Hypothetical nucleotide and amino acid sequences of *crtW*_*Fp*_ containing all identified SNPs (*crtW F. pelagi* MUT and CrtW AA-Seq. MUT) were aligned with the corresponding wild-type sequences (*crtW F. pelagi* WT and CrtW AA-Seq. WT) using *Clustal Omega* (Madeira et al., 2024) and *Boxshade* (URL: https://junli.netlify.app/apps/boxshade/). Generated SNPs as well as substitutions of amino acids were highlighted in the same colors as the employed protospacer sequence (HIS_1_ = red; HIS_1-2_ = purple; HIS_3_ = blue). In order to better classify the spatial localization of the substitutions within the enzyme, its histidine-rich regions were additionally marked (HIS I-III).

Regarding their amino acid sequence, histidine-rich regions (HIS-regions) conserved across multiple species (Supplementary Figure S2) are described for β-carotene ketolases (Ye et al., 2006). These regions are also typical for mechanistically related membrane desaturases (Shanklin et al., 1994) and are suspected to be involved in iron coordination (Arnold and Haymore, 1991) for the oxygen insertion process catalyzed by β-carotene ketolases (Bouvier et al., 1998). In accordance with this hypothesis, increased availability of iron also led to increased activity of the iron-dependent β-carotene ketolase from *F. pelagi* (CrtW_Fp_) (Meyer et al., 2025). Furthermore, alanine scanning mutagenesis revealed that six out of eight cytosolic histidine residues within these motifs of the β-carotene ketolase from *Paracoccus* sp. N81106 (CrtW_Pn_) are essential for its activity (Ye et al., 2006). Thus, it can be hypothesized that improving the steric accessibility of these histidine residues to iron through mutagenesis of adjacent sequence segments could yield enzyme variants with enhanced catalytic activity. Therefore, mutagenesis efforts focused on proximal sequences next to the histidine-rich regions (Supplementary Figure S2) (Figure 7). Three protospacers were independently cloned into gpEvolvR and the resulting constructs were transformed into *C. glutamicum* BETA4 Δ*actA*::P_Syn_-*crtW*_*Fp*_.

Following the mutagenesis protocol described, visual differences in colony pigmentation were observed (Supplementary Figure S4). Colonies showing color deviations from the initial strain were preferentially selected for curing of the gpEvolvR derivatives (Supplementary Figures S4,S5), and the resulting plasmid-free clones were scanned for documentation (Supplementary Figures S5,S6). To identify mutations responsible for altered pigmentation, both the *crtW*_*Fp*_ and the *crtY*_*Pa*_ gene were sequenced. The appearance of pink-colored colonies alongside red and orange variants (Supplementary Figure S4) suggested an impairment of CrtY_Pa_, which catalyzes the double cyclization of lycopene to β-carotene (Figure 1). HPLC analysis of mutant strains cultivated in 10 mL LB cultures for ∼24 h was performed for carotenoid quantification. The sequencing and HPLC results are shown in Supplementary Table S4.

Sequencing revealed various mutations within the *crtW*_*Fp*_ and *crtY*_*Pa*_ genes causing alterations within their encoded amino acid sequences compared to the reference strain (REF) *C. glutamicum* BETA4 Δ*actA*::P_Syn_-*crtW*_*Fp*_ (Figure 7). Certain mutations, such as the L233V substitution in *crtW*_*Fp*_ or even combination of mutations like in M24 and M25 arose multiple times independently. Notably, while mutants M6–8 carrying the CrtW_Fp_^L233V^ mutation exhibit similar carotenoid profiles, M26 shows a markedly different profile despite having identical *crtW*_*Fp*_ and *crtY*_*Pa*_ sequences, differing only in the sgRNA used for mutagenesis (Supplementary Table S4).

### 3.6 Production experiment with *crtW*_*Fp*_ mutant strains

To enhance expression and eliminate background effects from potential off-target mutations, *crtW*_*Fp*_ sequences from selected mutants with altered carotenoid profiles were cloned into pSH1. Cofactor limitations in production experiments were avoided by using CGXII_opt_ minimal medium supplemented with trace salts optimized for carotenoid production in *C. glutamicum* strains (Göttl et al., 2024).

**FIGURE 8:**
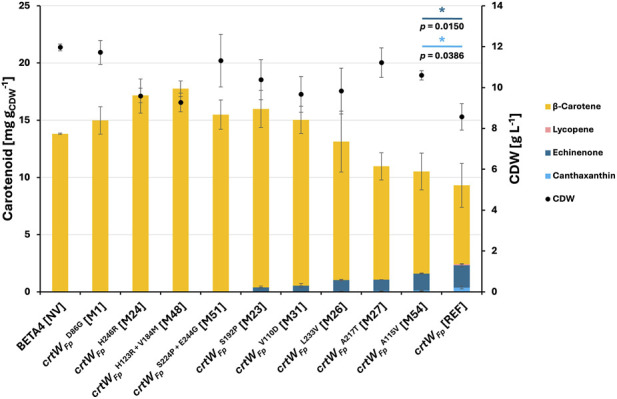
HPLC analysis and biomass concentration of BETA4 strains expressing mutant *crtW*_*Fp*_ variants. All *crtW*_*Fp*_ variants [MX] were expressed using the pSH1 backbone. The BETA4 harboring no vector [NV] and the BETA4 (pSH1-*crtW*_*Fp*_) expressing the non-mutated sequence of *crtW*_*Fp*_ [REF] served as references. Shown are the means of cell dry weight (CDW) and detected carotenoids relative to the CDW for 10 mL technical triplicates grown in CGXII_opt_ minimal medium supplemented with 40 g L^−1^ glucose (and kanamycin for pSH1 harboring strains) for 72 h with the respective standard deviation as Y-error. Statistical significance is based on a two-sided unpaired Student’s t-test (*: *p* ≤ 0.05; **: *p* ≤ 0.01). The strains were sorted in ascending order according to their mean concentrations of canthaxanthin.

Surprisingly, strains expressing a less active or even dysfunctional variant of the *crtW*_*Fp*_ gene (M1, M24, M48, M51, M23, and M31) tended to produce more β-carotene than the parental strain BETA4 (Figure 8; Supplementary Table S5). For instance, the mutants *crtW*_*Fp*_^H246R^ (M24) and *crtW*_*Fp*_^H123R+V184M^ (M48) produced approximately 24% or even 28% more β-carotene, respectively (Figure 8). However, episomal expression via the pSH1 backbone and the corresponding addition of kanamycin to the cultivation medium negatively affected biomass concentration, as the means of cell dry weights calculated from OD_600nm_ values were slightly reduced in these samples. In contrast, previous experiments conducted over a cultivation period of only 48 h observed the opposite effect (Figure 6).

Regarding the activity of *crtW*_*Fp*_, none of the mutated variants reached the titers of the reference sequence (REF) in respect to echinenone and canthaxanthin. Already for the second-best producer expressing the *crtW*_*Fp*_^A115V^ variant (M54), significantly lower levels of these CrtW_Fp_-derived products were detected by HPLC (Figure 8).

**FIGURE 9:**
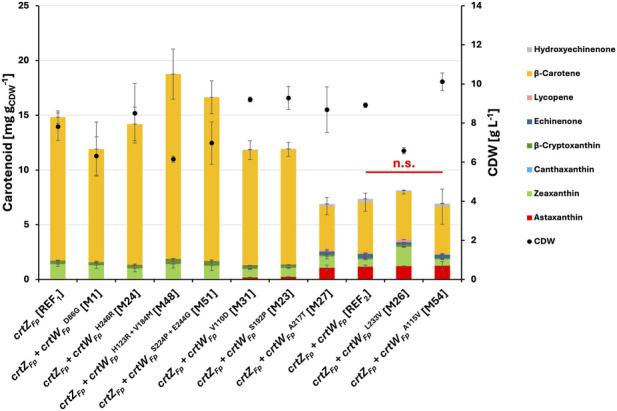
HPLC analysis and biomass concentration of BETA4 strains expressing mutant *crtW*_*Fp*_ variants together with *crtZ*_*Fp*_. All *crtW*_*Fp*_ variants [MX] were expressed using the pSH1 backbone, whereas *crtZ*_*Fp*_ was expressed using the pECXT-P_Syn_ backbone. The strains BETA4 (pECXT-P_Syn_-*crtZ*_*Fp*_) expressing the non-mutated sequence of *crtZ*_*Fp*_ [REF_1_] and BETA4 (pECXT-P_Syn_-*crtZ*_*Fp*_) (pSH1-*crtW*_*Fp*_) co-expressing the *crtZ*_*Fp*_ and *crtW*_*Fp*_ non-mutated sequences [REF_2_] served as references. Shown are the means of cell dry weight (CDW) and detected carotenoids relative to the CDW for 10 mL technical triplicates grown in CGXII_opt_ minimal medium supplemented with 40 g L^−1^ glucose and appropriate antibiotics for 72 h with the respective standard deviation as Y-error. The strains were sorted in ascending order according to their mean values for astaxanthin.

Next, transfer of the results regarding canthaxanthin production due to *crtW*_*Fp*_ mutations to astaxanthin production was assessed. Therefore, *crtW*_*Fp*_ strains were transformed with the pECXT-P_Syn_-*crtZ*_*Fp*_ plasmid (Figure 9).

When *crtZ*_*Fp*_ is additionally expressed, a similar production behavior can be observed (Figure 9). Variants of *crtW*_*Fp*_ that already showed comparable activity to the reference sequence in previous experiments (Figure 8), produced more astaxanthin in combination with *crtZ*_*Fp*_ expression than impaired variants (e.g., M31 and M23) and also showed a drastically reduced amount of total carotenoids (Figure 9). While the expression of the *crtW*_*Fp*_ variants M26 and M54 led to a slightly higher absolute mean value for astaxanthin compared to the reference sequence (Supplementary Table S6), this deviation is not statistically significant. In conclusion none of the *crtW*_*Fp*_ variants exhibited a significantly improved production behavior–neither when expressed alone (Figure 8; Supplementary Table S5) nor upon additional *crtZ*_*Fp*_ expression (Figure 9; Supplementary Table S6).

## 4 Discussion

In this work the EvolvR system was successfully transferred to *C. glutamicum* through design and construction of the gpEvolvR plasmid. Its mutagenic capabilities in *C. glutamicum* as well as *E. coli* on both genomic and episomal targets were demonstrated. The new gpEvolvR system serves as a dual-inducible adaptation of the EvolvR system and the temperature-sensitive pBL1_ts_ origin of replication enables its curability.

As a proof-of-principle mutagenesis of the pSC101 ori on the gpEvolvR plasmid demonstrated that the gpEvolvR plasmid is stable in *E. coli* DH5α via the pSC101 ori donated by pJYS3_easy cloning and demonstrated its ability to target itself for the diversification of nucleotides. As isolated mutants conferred an improved resistance to kanamycin as represented by the calculated minimal inhibitory concentration (MIC) it can be highlighted that the EvolvR plasmid system is an efficient tool for the evolution of nucleotide sequences for the scope of tailored phenotypes (Figure 5).

To validate the initial hypothesis that the mutations introduced by gpEvolvR caused an increase in PCN as indicated by improved antibiotic resistance, the exact PCNs for the wild-type plasmid and its mutant variants X1 and X9 need to be determined. This could be done with quantitative PCR (qPCR), relative quantification using the 2^−ΔΔCT^ method (Livak and Schmittgen, 2001) or droplet digital PCR (ddPCR) (Hindson et al., 2011).

Since the newly constructed gpEvolvR system was designed for *C. glutamicum* targeting of a chromosomal gene involved in carotenoid biosynthesis was chosen for its validation. As an experimental starting point an appropriate plate-based screening method was established for the visual discrimination of strains exhibiting different carotenoid profiles. Enhancement of carotenoid production by acetate addition likely results from an increased pyruvate and phosphoenolpyruvate (PEP) formation with boosted conversion into glyceraldehyde 3-phosphate (GAP) through gluconeogenesis (Wendisch et al., 2000) and thus making more primary carotenoid precursors available. Benefits from balanced co-utilization were observed for astaxanthin production already (Henke and Wendisch, 2019) and attribute to a synergistic effect of catabolism of glucose and acetate in *C. glutamicum* (Gerstmeir et al., 2003), which is known to lead to higher concentrations of glycolytic intermediates.

In principle, it can be assumed that random mutagenesis of a fragile, finely tuned system such as an enzyme will primarily result in dysfunctional variants (Arnold, 1996). This is especially true for the evolution of enzymes that do not confer a direct fitness gain like those involved in the carotenoid biosynthesis in *C. glutamicum*, which can be completely abolished by gene deletions with no effect on cell growth (Heider et al., 2012). Even with selection, random mutagenesis rarely leads directly to enhanced phenotypes (Kimura, 1983). The lack of improved CrtW variants likely reflects both the limited number of screened mutants and the restriction of mutagenesis to regions flanking essential histidine motifs. Consequently, the exploratory potential of the EvolvR system could be substantially enhanced by increasing library size through high-throughput screening approaches such as FACS and by targeting a broader range of sites across the *crtW* coding sequence.

Specifically for CrtW_Fp_ it is known that its iron-coordination (Arnold and Haymore, 1991; Ye et al., 2006) as well as the spatial arrangement of transmembrane and cytosolic domains (Shanklin et al., 1994; Ye et al., 2006) are important for its catalytic function. Thus, it is likely that mutations within the iron-coordinating HIS-regions (M24 and M48) or mutations presumably causing drastic changes to protein topology, such as the observed (M51 and M23) substitutions to proline (Glykos et al., 1999; Wychowaniec et al., 2024), led to impaired or even dysfunctional variants (Supplementary Tables S3,S4) as previously reported by (Ye et al., 2006).

However, studies investigating mutant CrtW sequences in different organisms showed partially contradictory behavior regarding the effect of sequence alternations in conserved and variable regions. For example, in *Paracoccus* sp. N81106 several mutations within the histidine motifs of CrtW were investigated with varying effects. Although, most mutations led to dysfunctional enzymes (e.g., corresponding positions to H123 and H246 in CrtW_Fp_; see Supplementary Figure S2), others only resulted in an impairment. On the other hand, the identified H219A variant was almost unaffected even though it is conserved within the third histidine motif, whereas the substituted histidine of the H184A variant does not belong to a reported histidine motif and still had a severe impact on the catalytic activity of CrtW (Ye et al., 2006). A similar behavior can be observed for proline substitutions within the CrtW sequences of other organisms. While the P12Q (P28 in CrtW_Fp_) substitution within CrtW of *Brevundimonas* sp. SD212 expressed in *E. coli* had an positive effect (Li et al., 2020), other substitutions of conserved prolines like P116A and P228A in *Paracoccus* sp. N81106 (P136 and P253 in CrtW_Fp_) had a rather negative effect on the catalytic activity of CrtW (Ye et al., 2006).

H123R (M48) and H246R (M24) are the only amino acid substitutions within CrtW_Fp_ that correspond to mutations described for the β-carotene ketolases of other organisms like *Sphingomonas melonis* DC18 (Tao et al., 2006), *Brevundimonas* sp. SD212 (Li et al., 2020), and *Paracoccus* sp. N81106 (Ye et al., 2006). For all other mutations, it can be assumed that they impair the catalytic activity of β-carotene ketolases e.g., based on steric hindrance that impedes the access of histidine motifs to iron ions (Li et al., 2020), or alter electron transport (Ye et al., 2006). Therefore, if protein crystal structures are not available, random mutagenesis represents an efficient way for the optimization of β-carotene ketolases within different organisms, emphasizing the importance of tools like gpEvolvR. In addition, as EvolvR enables targeted mutagenesis of defined loci, it would even benefit from recent advances in protein structure prediction using tools such as *AlphaFold* (Jumper et al., 2021) for the rationalization of suitable target sites.

Enzyme engineering is an incremental and labor-intensive process, characterized by iterative rounds of mutagenesis with subsequent extensive screening to gradually improve the enzymes functionality (Arnold, 1998). In fact, randomized mutagenesis performed by gpEvolvR, combined with high-throughput screenings follows this stepwise optimization paradigm. Notably, implementing such diversification in a continuous rather than discontinuous manner may accelerate evolutionary progress and increase the likelihood of achieving the desired outcome more efficiently. Applying a growth-coupled selective pressure such as light stress and reactive oxygen species might favor beneficial mutations in carotenogenesis (Bhosale, 2004) but could be technically difficult to apply through self-shading effects (Saccardo et al., 2022). Alternatively, as carotenoids have an effect on membrane fluidity the effect on altered sensitivity towards osmotic stress or heat might be exploited to create a selective pressure on carotenoid biosynthesis as already demonstrated in *Arthrobacter agilis* (Fong et al., 2001) and *Staphylococcus xylosus* (Seel et al., 2020).

Halperin et al. (2018) analyzed the targeting behavior of the EvolvR system in *E. coli* using the enCas9–PolI3M fusion and observed that mutations accumulated mainly within a defined region downstream of the nick site. This pattern was consistent with strand-displacement synthesis proceeding in the 5′ to 3′ direction on the strand complementary to the sgRNA. Although a broader region was analyzed in their study, the core mutational activity was concentrated within a narrower window close to the nick. In addition to PolI3M, Halperin et al. (2018) described a more error-prone variant, PolI5M, which showed strongly increased mutation frequencies directly adjacent to the nick but did not display a generally expanded mutational range or elevated mutation rates at more distant positions.

In our study, we applied enCas9–PolI5M in *C. glutamicum* rather than *E. coli* and observed a shifted and less confined distribution of mutations, including substitutions upstream of the predicted nick site as well as other loci. These results suggest that the mutational pattern of EvolvR might not exclusively be influenced by the employed nickase variant itself but also by the interplay with the host organism in which the system is implemented. We analyzed the chromosomally encoded CrtY_Pa_ as a reference gene to evaluate off-targeting. Loss-of-function mutants are identifiable by pinkish colonies that rely on the accumulation of lycopene based on a non-function lycopene-β-cyclase (CrtY_Pa_). The proline at position 190 in the amino acid sequence of CrtY_Pa_ was changed into an arginine (CrtY_Pa_^P190R^) in three independent cases (M4, M9, and M10) employing the protospacer HIS_1-2_ for mutagenesis. These three mutants exhibit nearly identical carotenoid profiles, whereas mutant M17, generated with the HIS_1_ protospacer-based sgRNA carries an alanine substitution at the same position (CrtY_Pa_^P190A^) and shows dramatically different carotenoid production patterns (Supplementary Table S4). Several factors complicated the characterization of *crtW*_*Fp*_ mutants: high redundancy of *crtW*_*Fp*_ mutations (Supplementary Table S4), potential unintended alterations to promoter sequences and *crtY*_*Pa*_, possible additional mutations at unknown genomic positions, and suboptimal conditions for CrtW_Fp_ and CrtY_Pa_ activity in LB medium. Therefore, a selection of mutants with unique mutations and diverse carotenoid profiles was examined in the context of the parental strain under optimized conditions.

The use of catalytically dead variants of the nuclease derived from *Streptococcus pyogenes* (dCas9) are well established in *C. glutamicum* for gene expression repression via CRISPR interference (CRISPRi) (Cleto et al., 2016; Gauttam et al., 2019; Göttl et al., 2021). Despite, the application of catalytically active nuclease (Cas9) or nickase (nCas9) was found to be toxic for *C. glutamicum* (Jiang et al., 2017). This toxicity was first attributed to non-specific PAM binding of Cas9 even in the absence of complementary crRNA, which would be particularly pronounced in *C. glutamicum* as it belongs to the actinobacteria phylum with characteristically high GC content, thus offering more than 14 PAMs per 100 bp of double-stranded DNA on average for potential off-target effects (Cho et al., 2017; Jiang et al., 2017). It later became apparent that Cas9 also binds to sequences proximal to the PAM with as little as four identical nucleotides, leading to undesired off-target effects in various bacteria (Rostain et al., 2023).

Curing the entire gpEvolvR plasmid prior to visual plate screening ensures a stable relationship between genotype and phenotype. As an alternative adapting of the *tetR/tetO* induction system with a reverse-acting tetracycline repressor variant carrying the substitutions G96E and L205S (Kamionka et al., 2004; Scholz et al., 2003) might be established in the future allowing the termination of the gene expression of the enCas9-PolI5M by aTc addition at any chosen timepoint.

Cas9-based off-target effects were found to be dependent on both nuclease and sgRNA expression levels (Hsu et al., 2013; Pattanayak et al., 2013). Corresponding attempts to reduce off-target activity induced toxicity by reducing the plasmid-borne induction in *C. glutamicum* (Peng et al., 2017; Yao et al., 2021) or by employing inducible chromosome-based expression (Wang B. et al., 2018) demonstrated to be successful. While chromosomal integration of the gpEvolvR system would defeat its purpose, reducing the expression of the enCas9-PolI5M fusion protein and the sgRNA could be achieved by titrating their inducers aTc and IPTG, respectively.

Alternative EvolvR enzymes could be designed e.g., with the recently released royalty free MAD7 (*Er*Cas12a) nuclease (Manus Bio Inc., Waltham, United States; formerly Inscripta). Originally derived from *Eubacterium rectale*, this Cas12a nuclease has been successfully employed for genome editing in mammalian (Wierson et al., 2019), microbial (Price et al., 2020), and plant cells (Lin et al., 2021). In fact, MAD7 has already been successfully used for engineering lycopene biosynthesis in *C. glutamicum*, increasing the yield by 102-fold compared to the parental strain (Zhan et al., 2024). Compared to *Fn*Cpf1, which relies on the PAM 5′-TTTN-3′ (Jiang et al., 2017), MAD7 depends on the PAM 5′-YTTN-3′, thus offering greater targeting flexibility in GC-rich strains like *C. glutamicum* (Zhan et al., 2024). As the EvolvR system depends on a nickase, the MAD7 nuclease could be subjected to an R1173A amino acid substitution conferring nickase activity (nMAD7) based on *As*Cpf1 crystal structure experiments performed by (Yamano et al., 2016).

## Data Availability

The original contributions presented in the study are included in the article/Supplementary Material, further inquiries can be directed to the corresponding author.
