# Supplementary material for: Design and validation of an inducible and curable EvolvR system for directed evolution in Corynebacterium glutamicum
Source: Front Bioeng Biotechnol. 2026 May 26;14:1827071. doi: 10.3389/fbioe.2026.1827071 (PMC13247561; doi:10.3389/fbioe.2026.1827071)
Supplement: Supplementary file 1 [file DataSheet1.docx]

Supplementary Material


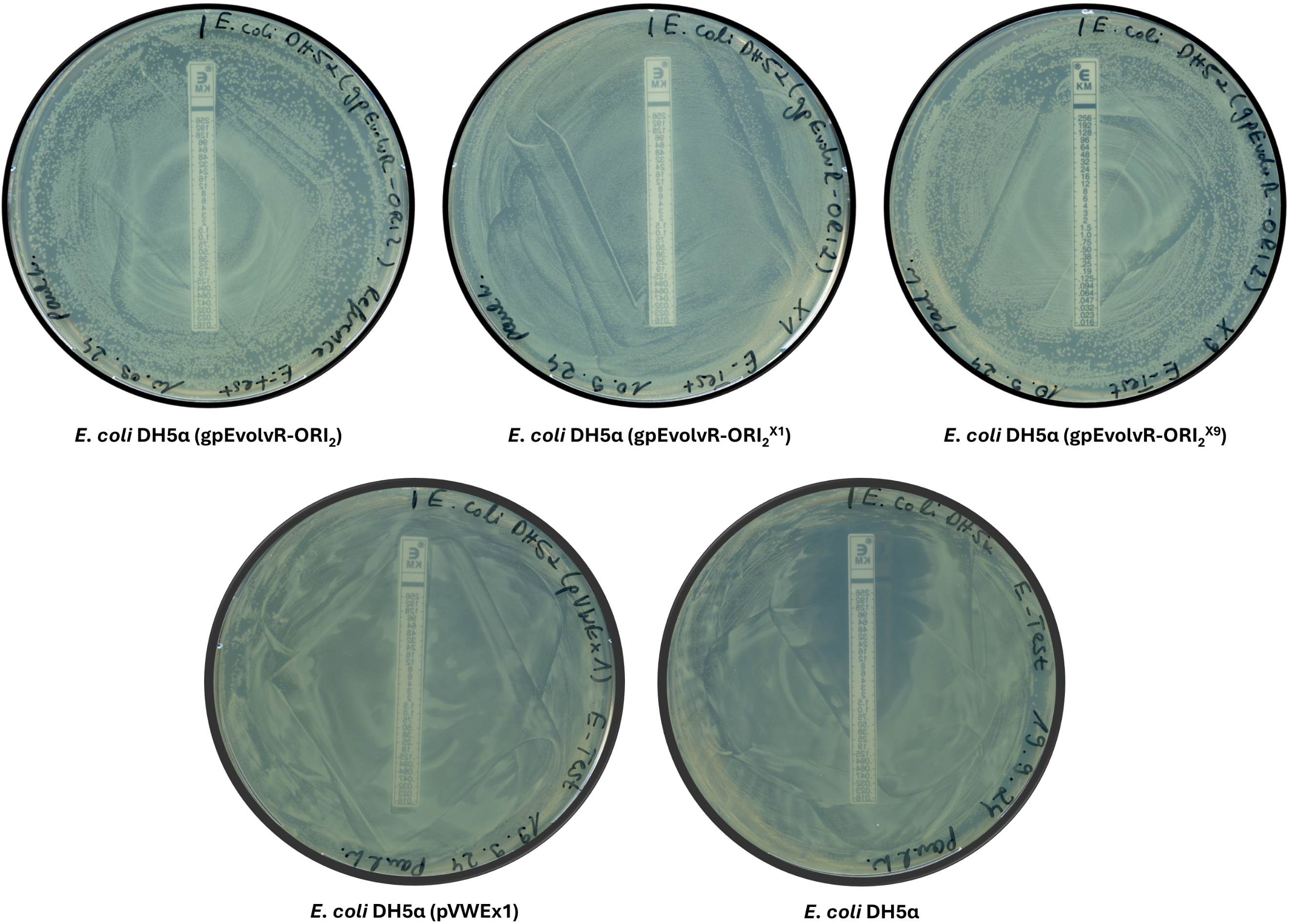


Figure S1: E-Test of the pSC101 ori mutants.

For references the wild-type plasmid gpEvolvR-ORI_2_ and pVWEx1 as another vector conferring resistance to kanamycin were tested. As a positive control the *E. coli* DH5α strain without any plasmid was used. Overnight cultures (LB medium + 1% glucose) of the strains were diluted to an OD_600nm_ of 0.1 and 100 µL of the cell suspension were spread on LB agar plates. On each plate a kanamycin E-Test stripe (Etest^®^ 0.016 – 256 µg mL^-1^, bioMérieux, LOT:1006035800) was placed followed by an incubation at 37 °C for 24 hours.


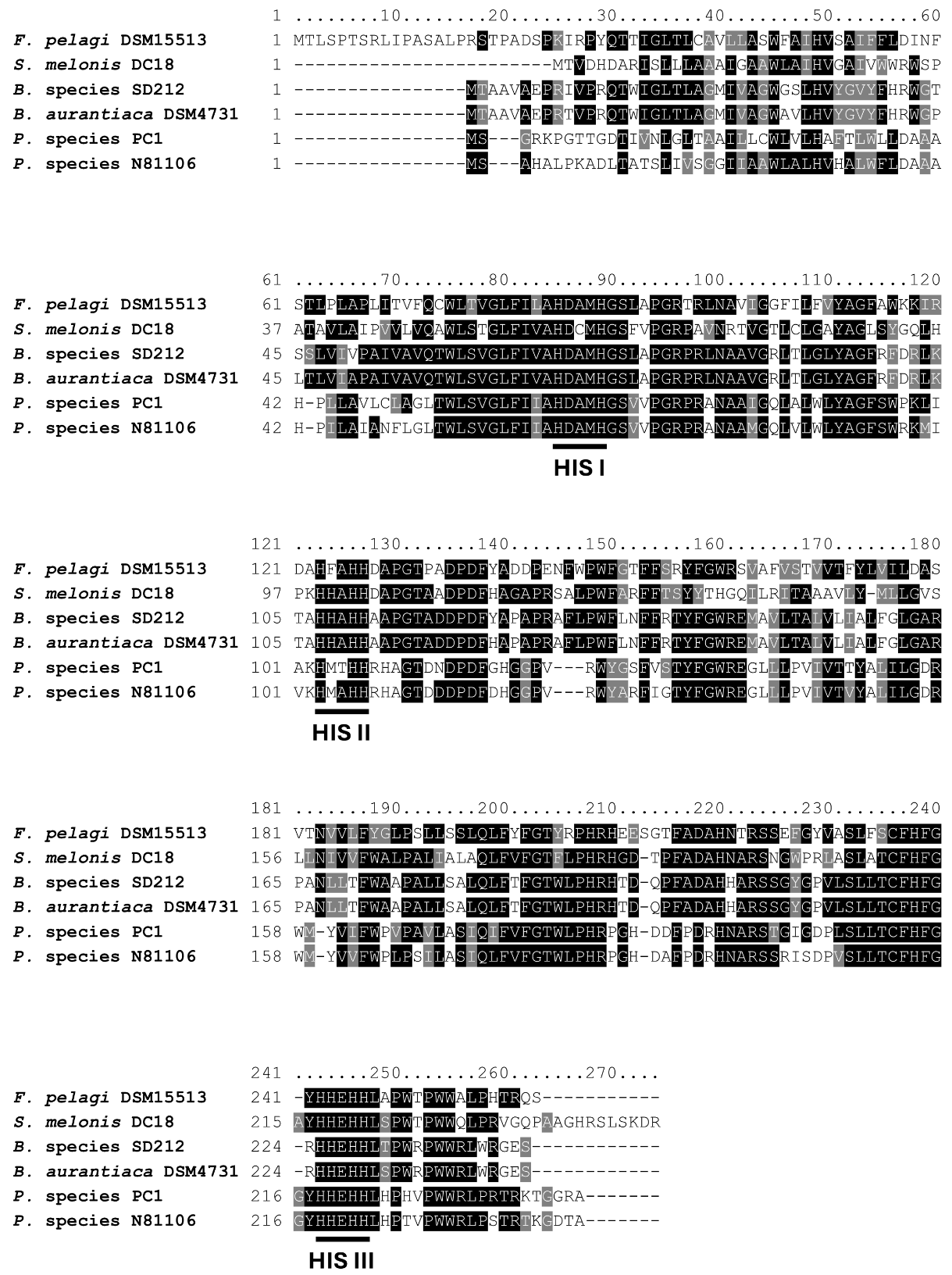


Figure S2: Similarity comparison of the amino acid sequences encoding for β-carotene ketolases (CrtW) in different organisms.

The sequences of *Fulvimarina pelagi* DSM15513 (GenBank accession number BAT30875), *Sphingomonas melonis*DC18 (GenBank accession number ABD61226), *Brevundimonas* species SD212 (GenBank accession number BAD99406), *Brevundimonas aurantiaca*DSM4731 (GenBank accession number AAN86030), *Paracoccus*species PC1 (GenBank accession number BAA09596) and *Paracoccus*species N81106 (GenBank accession number BAE47465) were aligned using *Clustal Omega* (Madeira et al., 2024) and identical or similar amino acids highlighted using *Boxshade* (URL:https://junli.netlify.app/apps/boxshade/). Conserved histidine motifs (HIS I-III) are indicated.


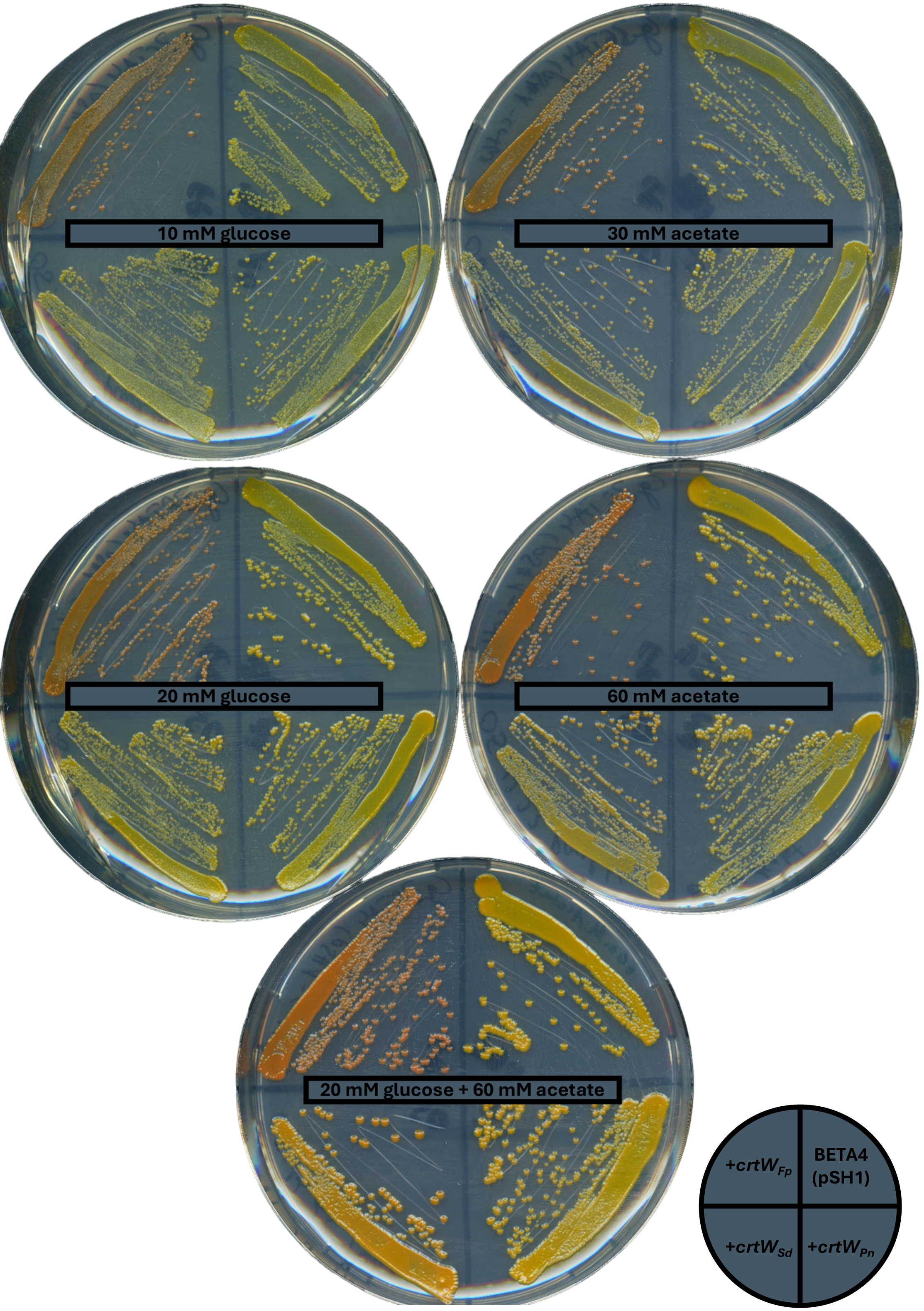


Figure S3: Pigmentation of *C. glutamicum* BETA4 strains expressing *crtW* variants on CGXII_opt/Km25_ agar plates containing different carbon source compositions.

As carbon sources, glucose (10 mM and 20 mM) as well as its carbon-equivalent concentrations of acetate (30 mM and 60 mM) and a combination of both (20 mM glucose and 60 mM acetate) were used. The corresponding empty vector strain *C. glutamicum* BETA4 (pSH1) served as a reference to the strains expressing *crtW* variants. Plates were incubated at 30 °C for 2 days followed by incubation at room temperature for another 4 days.


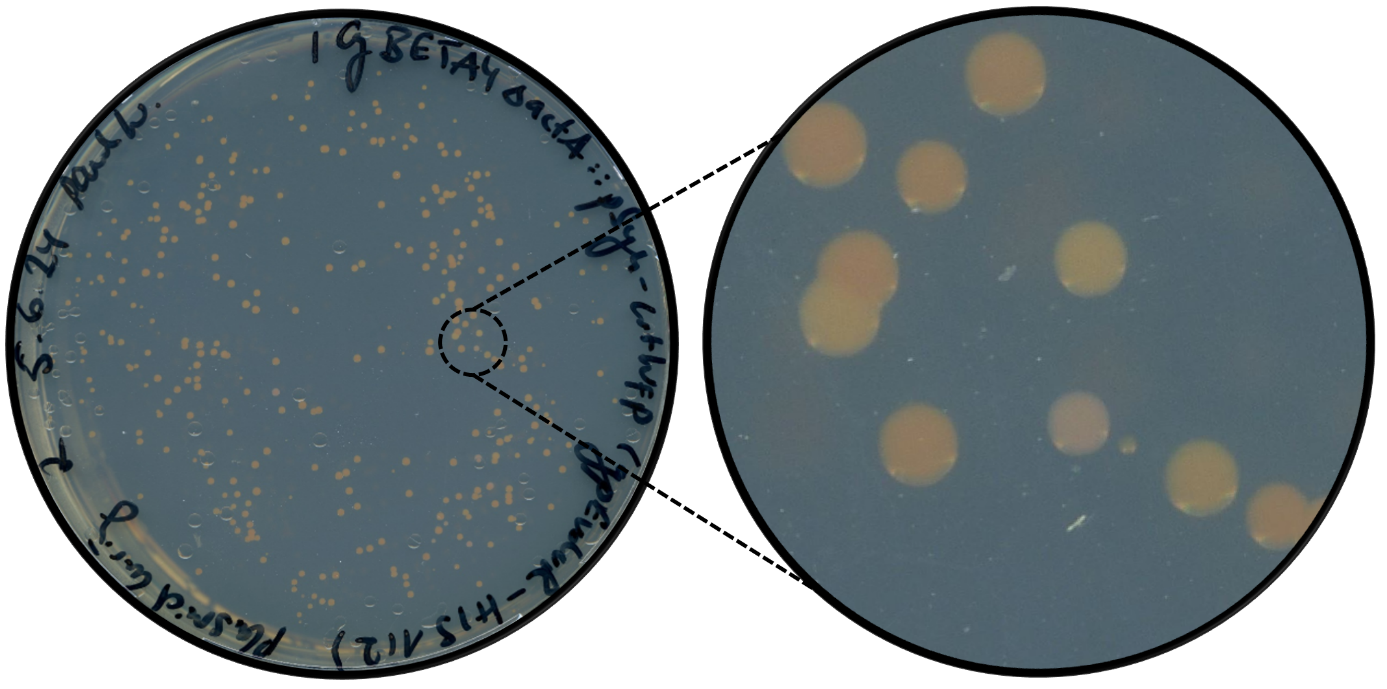


Figure S4: Pigmentation of *C. glutamicum* BETA4 Δ*actA*::P_Syn_-*crtW_Fp_* (gpEvolvR-HIS_1-2_) cell colonies after mutagenesis.

For plasmid curing, 100 µL of an OD_600nm_ 0.0001 dilution were spread onto CGXII_opt_ agar plates supplemented with 60 mM acetate and 20 mM glucose and incubated at 37 °C for 48 hours.


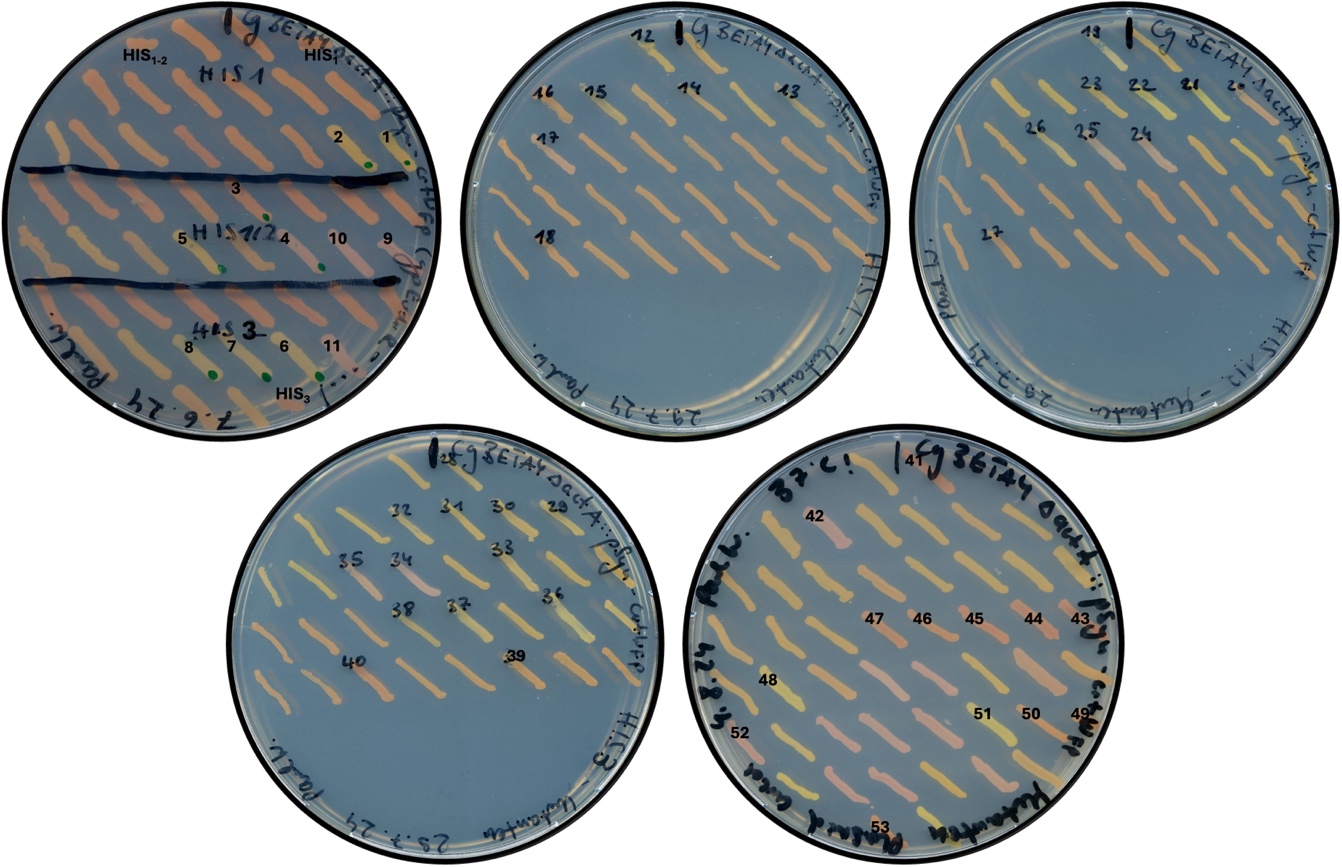


Figure S5: Phenotypes of plasmid-cured *crtW_Fp_* mutants generated using gpEvolvR derivatives in *C. glutamicum* BETA4 Δ*actA*::P_Syn_-*crtW_Fp_*.

Mutagenesis and plasmid curing were performed as described in methods section. Selected clones were numbered for identification, while parental strains containing the respective gpEvolvR derivatives prior to mutagenesis served as visual references for phenotypic changes (top left plate).


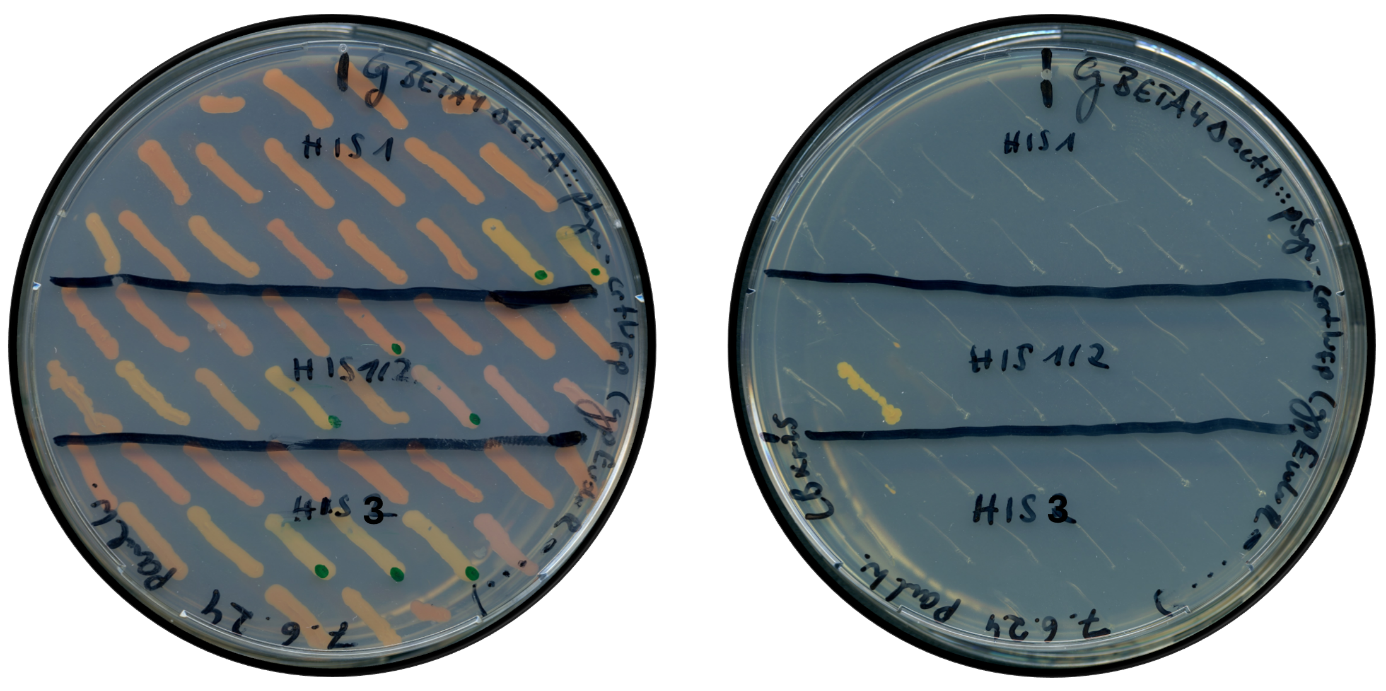


Figure S6: Results of gpEvolvR curing via the temperature sensitive pBL1_ts_ origin of replication.

Plasmid cured clones were incubated on CGXII_opt_ agar plates supplemented with 20 mM glucose and 60 mM acetate for 72 hours, containing either no antibiotics (left plate) or 25 µg mL^-1^ kanamycin (right plate). Clones that have been successfully cured from gpEvolvR also lost its *kanR* resistance cassette and therefore the ability to grow in the presence of kanamycin.

Table S1: Oligonucleotides used in this study.

| ID | Function | Sequence |
| --- | --- | --- |
| 049P | Amplification of the upstream flank region for integration of pK19*mobsacB* based constructs into the *actA* (cg2840) locus (fw) | GCTATGACCATGATTACGCCAAGCTTGCATGCCTGCAGGTCGACTCTAGAGTGCGTCCAGCATCCAAGTG |
| 050P | Amplification of the upstream flank region for integration of pK19*mobsacB* into the *actA* (cg2840) locus for the construction of pK19*mobsacB*-*P_Syn_-crtW_Fp_* (rv) | CGAATTCCATGGAACCATTATAACACAGATTCAAATTAATGTCAATGAATACCTCCTCAGGTAATCGGAC |
| 051P | Sequencing and amplification of the *crtW_Fp_* gene together with the *P_Syn_* promoter from pECXT-*P_Syn_-crtW_Fp_* for the construction of pK19*mobsacB*-*P_Syn_-crtW_Fp_* (fw) | GAAAAAGTCCGATTACCTGAGGAGGTATTCATTGACATTAATTTGAATCTGTGTTATAATGGTTCCATG |
| 052P | Sequencing and amplification of the *crtW_Fp_* gene for the construction of pK19*mobsacB* based constructs (rv) | CCGCCGCCTCGAAATGTTGTCGAGGCGGCGGTTTCTAAACCAAGAAAAAACTTAGGACTGGCGAGTATGCGG |
| 053P | Amplification of the downstream flank region for integration of pK19*mobsacB* based constructs carrying the *crtW_Fp_* gene into the *actA* (cg2840) locus (fw) | GGACGCCTTGGTGGGCTCTGCCGCATACTCGCCAGTCCTAAGTTTTTTCTTGGTTTAGAAACCGCCGC |
| 054P | Amplification of the downstream flank region for integration of pK19*mobsacB* based constructs into the *actA* (cg2840) locus (rv) | GACGTTGTAAAACGACGGCCAGTGAATTCGAGCTCGGTACCCGGGGATCGTGCATCAGAGTGACGGGAAC |
| 055P | cPCR of the *actA* (cg2840) locus for verification of the respective integrations (fw) | CGCAGGATGATTTGGGTAACTTCG |
| 056P | cPCR of the *actA* (cg2840) locus for verification of the respective integrations (rv) | GAAGGGGCACATGATGTCGC |
| 057P | Amplification of the upstream flank region for integration of pK19*mobsacB* into the *actA* (cg2840) locus for the construction of pK19*mobsacB*-*P_H36_-crtW_Fp_* (rv) | CGTTAATATTCCCCCGTTTAGGGCACCAGATAGAGGTACCCAGCTTTTGTGAATACCTCCTCAGGTAATCGGACTTTTTC |
| 058P | Amplification of the *crtW_Fp_* gene and addition of the *P_H36_* promoter sequence for the construction of pK19*mobsacB*-*P_H36_-crtW_Fp_* (fw_1_) | CGGGGGAATATTAACGGGCCCAGGGTGGTCGCACCTTGGTTGGTAGGAGTAGCATGCATGGAATTCGAGCTCGGTACCC |
| 059P | Amplification of the *crtW_Fp_* gene and addition of the *P_H36_* promoter sequence for the construction of pK19*mobsacB*-*P_H36_-crtW_Fp_* (fw_2_) | CCTGAGGAGGTATTCACAAAAGCTGGGTACCTCTATCTGGTGCCCTAAACGGGGGAATATTAACGGGCC |
| 060P | Amplification of the upstream flank region for integration of pK19*mobsacB* into the *actA* (cg2840) locus for the construction of pK19*mobsacB*-*P_tuf_-crtW_Fp_* (rv) | CAAACTACCAGCTACCCTGTGGACATTCGCAGGGTAACGGCCATGAATACCTCCTCAGGTAATCGGAC |
| 061P | Amplification of the *crtW_Fp_* gene together with the *P_tuf_* promoter from pECXT-*P_tuf_-crtW_Fp_* for the construction of pK19*mobsacB*-*P_tuf_-crtW_Fp_* (fw) | GCAGTGCGGGAAAAATTTGAAAAAGTCCGATTACCTGAGGAGGTATTCATGGCCGTTACCCTGCG |
| 063P | Sequencing of the upstream flank region within the *actA* (cg2840) locus for verification of respective integrations (fw) | CGGGCATTTCCTGTCACGATG |
| 1023 | Sequencing of the downstream flank region within the *actA* (cg2840) locus for verification of respective integrations (rv) | CTGGCTCGGAGCATTCTC |
| 037P | Amplification of the *tetR* regulatory system and a *Sal*I and *Pst*I cured fragment of *enCas9* from gene synthesis for construction of pSH1-EvolvR (fw) | GGACATACAAAGCTTGCATGCCTGCAGGTCGACTCTAGAGTTAAGACCCACTTTCACATTTAA GTTGTTTTTCTAATCC |
| 038P | Amplification of the *tetR* regulatory system and a *Sal*I and *Pst*I cured fragment of *enCas9* from gene synthesis for construction of pSH1-EvolvR (rv) | GGTAGATAAGCCGGAGGTCTGCCTTGTCGGTTGAGTCCACCAGCTTTTTGCGAAGATGGTAGATGGTTGG |
| 039P | Amplification of a *Pst*I cured fragment of *enCas9*-*polI5M* from pEvolvR-*enCas9*-*polI5M* for construction of pSH1-EvolvR (fw) | GGCCTATCACGAGAAATACCCAACCATCTACCATCTTCGCAAAAAGCTGGTGGACTCAACCGACAAGG |
| 040P | Amplification of a *Pst*I cured fragment of *enCas9*-*polI5M* from pEvolvR-*enCas9*-*polI5M* for construction of pSH1-EvolvR (rv) | GGCTTGATACCTTGCTTCTCGAACAGGATAGTCTGGAGTTGTTTCGTAGATGACAGATTGAAC |
| 041P | Amplification of a fragment of *polI5M* with the *L3S2P55* terminator from pEvolvR-*enCas9*-*polI5M* for construction of pSH1-EvolvR (fw) | CGCTGGCGAAGAGTTCAATCTGTCATCTACGAAACAACTCCAGACTATCCTGTTCGAGAAGCAAGG |
| 042P | Amplification of a fragment of *polI5M* with the *L3S2P55* terminator from pEvolvR-*enCas9*-*polI5M* for construction of pSH1-EvolvR (rv) | CGGCCAGTGAATTCGAGCTCGGTACCCGGGGATCGGACCAAAACGAAAAAAGACGCTTTTC |
| 043P | Amplification of the subcloned *Pst*I and *Sal*I cured *enCas9*-*polI5M* from pSH1-EvolvR for construction of gpEvolvR (fw) | CGCCTTCTTGACGAGTTCTTCTGAGCGGGACTCTGTTAAGACCCACTTTCACATTTAAGTTGTTTTTCTAATCC |
| 044P | Amplification of the subcloned *Pst*I and *Sal*I cured *enCas9*-*polI5M* from pSH1-EvolvR for construction of gpEvolvR (rv) | CGCCGAAACGTTTGGTGGCGGGACCAGTGACGAAGGCTTGAGGACCAAAACGAAAAAAGACGCTTTTC |
| 045P | Amplification of the *lacI^q^* regulatory system and the sgRNA scaffold from pS_dCas9 for construction of gpEvolvR (fw) | CCAAAGACGAACAATAAGACGCTGAAAAGCGTCTTTTTTCGTTTTGGTCCTCAAGCCTTCGTCACTGGTCC |
| 046P | Amplification of the *lacI^q^* regulatory system and the sgRNA scaffold from pS_dCas9 for construction of gpEvolvR (rv) | GGCTAAATACGGAAGGATCTGAGGTTCTTATGGCTCTTGTATCTATACCCGGGGATCCTCTAGAGTC |
| 047P | Amplification of *kanR*, pBL1_ts_, pSC101 ori and *repA101* from pJYS3_easy cloning for construction of gpEvolvR (fw) | CGGTGCTTTTTTCGTCGACTCTAGAGGATCCCCGGGTATAGATACAAGAGCCATAAGAACCTCAGATCC |
| 048P | Amplification of *kanR*, pBL1_ts_, pSC101 ori and *repA101* from pJYS3_easy cloning for construction of gpEvolvR (rv) | CATATGCGGATTAGAAAAACAACTTAAATGTGAAAGTGGGTCTTAACAGAGTCCCGCTCAGAAGAACTC |
| 013P | Sequencing of gpEvolvR (fw) | CCTTTCTTCTTTAGCGACTTGATGCTC |
| 014P | Sequencing of gpEvolvR (fw) | GGACTAATAGCGTCGGGTGGG |
| 015P | Sequencing of gpEvolvR (fw) | GTCAAAGAGCCGCAGACTTGAG |
| 016P | Sequencing of gpEvolvR (fw) | CAAGAGGACTTCTACCCCTTTCTTAAGG |
| 017P | Sequencing of gpEvolvR (fw) | TTAAGACCTACGCCCATCTGTTCG |
| 018P | Sequencing of gpEvolvR (fw) | CAGAGCTTCCTCGCAGACG |
| 019P | Sequencing of gpEvolvR (fw) | CACCCTTGCAAACGGTGAGATC |
| 020P | Sequencing of gpEvolvR (fw) | TTTGTGGAGCAGCATAAGCATTATCTG |
| 021P | Sequencing of gpEvolvR (fw) | CTATAAGTCTCACCGTCCTCCGATG |
| 022P | Sequencing of gpEvolvR (fw) | GCGTTGGACCGCCGAC |
| 023P | Sequencing of gpEvolvR (fw) | CTGGAGGAAGCTGGTCGC |
| 024P | Sequencing of gpEvolvR (fw) | GAAGACTACGTTATCGTTTCTGCTGATTATTCTC |
| 025P | Sequencing of gpEvolvR (fw) | GCTTAGACGTTCCGCTGCTG |
| 026P | Sequencing of gpEvolvR (fw) | AGCCAGCCAGACGCAG |
| 027P | Sequencing of gpEvolvR (fw); cPCR of the pSC101 ori from gpEvolvR constructs and sequencing of the amplicon | GAATTGACTCTCTTCCGGGCG |
| 028P | Sequencing of gpEvolvR (fw) | TTGAGATCATGCTTACTTTGCATGTCAC |
| 029P | Sequencing of gpEvolvR (fw) | CCACAGTTCTCGTCATCAGCTC |
| 030P | Sequencing of gpEvolvR (fw) | AAGCTCGGGCAAATCGCTG |
| 031P | Sequencing of gpEvolvR (fw) | CAGAACCGCGTCCAAGTCC |
| 032P | Sequencing of gpEvolvR (fw) | GCAGACCAGCGGGAAAACATG |
| 033P | Sequencing of gpEvolvR (fw) | TGCGCAGTGCGAGTTTGTG |
| 034P | Sequencing of gpEvolvR (fw) | CACAAGCCACCATCGAAGCAC |
| 035P | Sequencing of gpEvolvR (fw) | GGATGAATGTCAGCTACTGGGCTATC |
| 036P | Sequencing of gpEvolvR (fw) | CGGCTGCATACGCTTGATCC |
| 091P | Sequencing of gpEvolvR (rv) | CAACTTAAATGTGAAAGTGGGTC |
| 092P | Sequencing of gpEvolvR (rv) | CTCTGCCAGGTCGAAATTAGAC |
| 093P | Sequencing of gpEvolvR (rv) | CTTGAATGCGGCAGGGGC |
| 094P | Sequencing of gpEvolvR (rv) | CGCAGACATACCGTAGATTAAGCCG |
| 095P | Sequencing of gpEvolvR (rv) | GACAGCTCATGTTATATCCCGCCG |
| 096P | Sequencing of gpEvolvR (rv) | CAACGCGTCAGTGGGCTG |
| 097P | Sequencing of gpEvolvR (rv); cPCR of the pSC101 ori from gpEvolvR constructs and sequencing of the amplicon | GCCCGTGACGGGCTTTTC |
| 098P | Sequencing of gpEvolvR (rv) | CATGCGCATGCGTAGCAAAC |
| 099P | Sequencing of gpEvolvR (rv) | CGGCTTCTTCGTCTAGGGC |
| 100P | Sequencing of gpEvolvR (rv) | CCAGAAAAGCGGCCATTTTCC |
| 068P | First complementary oligonucleotide for the annealing process yielding an integrable protospacer (ORI_2_) for construction of  gpEvolvR-ORI_2_ targeting the pSC101 ori | AAATTCTGCTAGACCTTTGCGTTTTAGAGCTAGAAATAGCAAGTTAAAAT |
| 069P | Second complementary oligonucleotide for the annealing process yielding an integrable protospacer (ORI_2_) for construction of  gpEvolvR-ORI_2_ targeting the pSC101 ori | GCAAAGGTCTAGCAGAATTTTGCAGGCATGCAAGCTTGGCGTAATCATGG |
| 083P | First complementary oligonucleotide for the annealing process yielding an integrable protospacer (HIS_1_) for construction of  gpEvolvR-HIS_1_ targeting the *crtW_Fp_* | CATGCATGGCGTCGTGAGCCGTTTTAGAGCTAGAAATAGCAAGTTAAAAT |
| 084P | Second complementary oligonucleotide for the annealing process yielding an integrable protospacer (HIS_1_) for construction of  gpEvolvR-HIS_1_ | GGCTCACGACGCCATGCATGTGCAGGCATGCAAGCTTGGCGTAATCATGG |
| 085P | First complementary oligonucleotide for the annealing process yielding an integrable protospacer (HIS_1-2_) for construction of  gpEvolvR-HIS_1-2_ targeting the *crtW_Fp_* | CAAATCCCGCGTAGACGAACGTTTTAGAGCTAGAAATAGCAAGTTAAAAT |
| 086P | Second complementary oligonucleotide for the annealing process yielding an integrable protospacer (HIS_1-2_) for construction of  gpEvolvR-HIS_1-2_ | GTTCGTCTACGCGGGATTTGTGCAGGCATGCAAGCTTGGCGTAATCATGG |
| 089P | First complementary oligonucleotide for the annealing process yielding an integrable protospacer (HIS_3_) for construction of  gpEvolvR-HIS_3_ targeting the *crtW_Fp_* | GCTCATGGTGGTAGCCAAAAGTTTTAGAGCTAGAAATAGCAAGTTAAAAT |
| 090P | Second complementary oligonucleotide for the annealing process yielding an integrable protospacer (HIS_3_) for construction of  gpEvolvR-HIS_3_ targeting the *crtW_Fp_* | TTTTGGCTACCACCATGAGCTGCAGGCATGCAAGCTTGGCGTAATCATGG |
| vgai | Amplification and sequencing of the sgRNA scaffold of gpEvolvR for verification of protospacer integration (fw) | GTATGGCTGTGCAGGTCGTAAA |
| 076P | Amplification and sequencing of the sgRNA scaffold of gpEvolvR for verification of protospacer integration (rv) | TACCCGGGGATCCTCTAGAGTC |
| 103P | Amplification of *crtW_Fp_* variants from *actA* (cg2840) locus for integration into pSH1 (fw) | GGAGGACATACAAAGCTTGCATGCCTGCAGGTCGACTCTAGAGGGAGGCGACTCTATTCGAGACTACC |
| 102P | Amplification of *crtW_Fp_* variants from *actA* (cg2840) locus for integration into pSH1 (rv) | GGCTGTAAAACGACGGCCAGTGAATTCGAGCTCGGTACCCGGGGATCTTAGGACTGGCGAGTATGCGG |
| 1129 | cPCR and sequencing of pSH1 derivatives (fw) | ACCGGCTCCAGATTTATCAG |
| 1135 | cPCR and sequencing of pSH1 derivatives (rv) | TACTGCCGCCAGGCAAATTC |
| V209 | Sequencing of *crtW_Fp_* (fw) | GGTTCCGGCGGTGGCGGTTCCGGCGGTGGCGGTTCCGGCGGTCCAGGTTCCACCCTCAGCCCAACCTCAC |
| V201 | Sequencing of *crtW_Fp_* (fw) | GGGTTCATCCTGTTCGTCTACGC |
| V680 | Amplification of *crtY_Pa_* from the *C. glutamicum* BETA4 genome and sequencing (fw) | GCACTAGTCGATCTTGAAACTACCC |
| V099 | Amplification of *crtY_Pa_* from the *C. glutamicum* BETA4 genome and sequencing (rv) | GCAGGTCGACTCTAGAGGATCTTAACGATGAGTCGTCATAATGGCTTGC |
| V100 | Sequencing of *crtY_Pa_* (fw) | CGTCTCCCATTATCATGGATGCC |
| V101 | Sequencing of *crtY_Pa_* (fw) | GCAGCGTTTTTATGGTTTACCTGAAG |

Table S2: List of features and their function in the novel gpEvolvR plasmid.

| Donor | Feature | Function |
| --- | --- | --- |
| Gene synthesis | *tetR* and *tetA*/*tetO* | Regulation of *enCas9-polI5M* expression by aTc induction. (Hillen & Berens, 1994) |
| pEvolvR-*enCas9*-*polI5M* | *enCas9* | Nickase derived from *Streptococcus pyogenes* Cas9 by introduction of a D10A mutation which causes nicking of the strand complementary to the sgRNA and K848A, K1003A and R1060A for the reduction of nonspecific DNA affinity. (Halperin et al., 2018; Slaymaker et al., 2016) |
| pEvolvR-*enCas9*-*polI5M* | *polI5M* | Error-prone DNA polymerase derived from *Escherichia coli* DNA polymerase I by introduction of D424A, I709N, A759R, F742Y and P796H mutations. (Halperin et al., 2018) |
| pJYS3_easy cloning | *kanR* or *nptII* | Confers antibiotic resistance to kanamycin, neomycin and geneticin (G418). It serves as a selection marker and supports plasmid maintenance. (Beck et al., 1982) |
| pJYS3_easy cloning | pBL1_ts_ | Temperature sensitive replicon based on the pBL1 ori from *Corynebacterium glutamicum* ATCC13869 cryptic plasmid pBL1 mutagenized by hydroxylamine treatment to maintain stable at 25°C but not at > 34 °C. Replication via rolling circle model. (J. Nakamura et al., 2006) |
| pJYS3_easy cloning | pSC101 ori | Low-copy replication origin for *Escherichia coli* from the pSC101 plasmid that requires the RepA101 protein. (Cohen et al., 1973; Yamaguchi & Yamaguchi, 1984) |
| pJYS3_easy cloning | *repA101* | Encodes for the RepA101 protein needed for replication with the pSC101 ori. (Cohen et al., 1973; Yamaguchi & Yamaguchi, 1984) |
| pS_dCas9 | *lacI^q^* | Expression of the lactose repressor gene *lacI* regulating sgRNA expression by IPTG induction. A single base exchange in the -35 promoter region (I^q^) resulted in a 10-fold enhancement of *lacI* expression. (Calos, 1978) |
| pS_dCas9 | sgRNA scaffold | *Pst*I recognition site in front of an Cas9 handle followed by a terminator sequence from *Streptococcus pyogenes* allowing convenient integration of protospacer DNA via Gibson assembly. Due to an *Sal*I recognition site following the terminator, multiple scaffold integration for multiplexing is possible. (Gauttam et al., 2019; Göttl et al., 2021) |

Table S3: Cell dry weights (CDW) [g L^-1^] and carotenoids [mg g_CDW_^-1^] produced by the *C. glutamicum* BETA4 Δ*actA*::*crtW_Fp_* integration strains with *crtW_Fp_* under the control of promoters of different strengths.

Mean values and standard deviations correspond to Figure 6B.

| Strain | CDW | Lycopene | β-carotene | | Echinenone | Canthaxanthin | Total carotenoids |
| --- | --- | --- | --- | --- | --- | --- | --- |
| BETA4 | 10.27 ±0.94 | 0.09 ±0.01 | 18.33 ±1.49 | N/A | | N/A | 18.42 ±1.50 |
| BETA4 Δ*actA*::P_H36_-*crtW_Fp_* | 11.72 ±0.71 | 0.07 ±0.00 | 14.99 ±0.86 | 0.02 ±0.00 | | N/A | 15.08 ±0.86 |
| BETA4 Δ*actA*::P_tuf_-*crtW_Fp_* | 11.58 ±0.74 | 0.07 ±0.00 | 13.76 ±0.81 | 0.47 ±0.04 | | 0.01 ±0.00 | 14.31 ±0.85 |
| BETA4 Δ*actA*::P_Syn_-*crtW_Fp_* | 12.23 ±0.21 | 0.06 ±0.01 | 10.23 ±1.15 | 0.65 ±0.21 | | 0.02 ±0.01 | 10.96 ±1.37 |
| BETA (pSH1-*crtW_Fp_*) | 12.67 ±0.21 | 0.17 ±0.02 | 1.65 ±0.25 | 0.58 ±0.15 | | 1.02 ±0.06 | 3.42 ±0.37 |

Table S4: Summary of mutant strain analysis.

Shown are the changes within the amino acid sequence of CrtW_Fp_ and CrtY_Pa_ revealed by sanger sequencing of the respective encoding genes, as well as the carotenoid content of mutant strains cultivated for 24 hours in LB medium quantified via HPLC in mg g_CDW_^-1^. Also, the respective protospacer within the sgRNA used in the mutagenic process is specified. Unclear sequencing results were labeled with “N/A”.

| ID | sgRNA | CrtW_Fp_ | CrtY_Pa_ | Lycopene | β-carotene | Echinenone | Canthaxanthin |
| --- | --- | --- | --- | --- | --- | --- | --- |
| REF | --- | --- | --- | 0.00 | 1.71 | 0.67 | 0.03 |
| M1 | HIS_1_ | D86G | --- | 0.00 | 5.35 | 0.01 | 0.00 |
| M2 | HIS_1_ | D86G | --- | 0.00 | 3.07 | 0.00 | 0.00 |
| M3 | HIS_1-2_ | --- | --- | 0.04 | 1.30 | 0.76 | 0.10 |
| M4 | HIS_1-2_ | --- | P190R | 1.12 | 0.00 | 0.00 | 0.00 |
| M5 | HIS_1-2_ | Deletion at 236bp; early stop-codon | --- | 0.03 | 4.24 | 0.00 | 0.00 |
| M6 | HIS_3_ | L233V | --- | 0.00 | 0.38 | 0.28 | 0.02 |
| M7 | HIS_3_ | L233V | --- | 0.00 | 0.33 | 0.20 | 0.01 |
| M8 | HIS_3_ | L233V | --- | 0.00 | 0.39 | 0.26 | 0.02 |
| M9 | HIS_1-2_ | --- | P190R | 1.14 | 0.00 | 0.00 | 0.00 |
| M10 | HIS_1-2_ | --- | P190R | 1.13 | 0.00 | 0.00 | 0.00 |
| M11 | HIS_3_ | H246R | L84P | 1.31 | 0.00 | 0.00 | 0.00 |
| M12 | HIS_1_ | D86G | --- | 0.00 | 9.33 | 0.00 | 0.00 |
| M13 | HIS_1_ | --- | --- | 0.09 | 2.24 | 1.41 | 0.20 |
| M14 | HIS_1_ | --- | --- | 0.00 | 1.14 | 1.03 | 0.20 |
| M15 | HIS_1_ | --- | --- | 0.07 | 2.05 | 1.42 | 0.14 |
| M16 | HIS_1_ | --- | --- | 0.08 | 1.25 | 1.21 | 0.28 |
| M17 | HIS_1_ | --- | P190A | 1.72 | 1.71 | 0.06 | 0.01 |
| M18 | HIS_1_ | --- | --- | 0.09 | 2.20 | 1.48 | 0.13 |
| M19 | HIS_1-2_ | --- | --- | 0.00 | 0.00 | 0.13 | 0.16 |
| M20 | HIS_1-2_ | V40A L233L | V177A | 1.39 | 1.62 | 0.07 | 0.02 |
| M21 | HIS_1-2_ | Deletion at 717bp; stop-codon removed | --- | 0.00 | 6.02 | 0.00 | 0.00 |
| M22 | HIS_1-2_ | Deletion at 717bp; stop-codon removed | --- | 0.00 | 5.64 | 0.00 | 0.00 |
| M23 | HIS_1-2_ | S192P | --- | 0.00 | 4.82 | 0.32 | 0.00 |
| M24 | HIS_1-2_ | H246R | L84P | 1.76 | 1.65 | 0.00 | 0.00 |
| M25 | HIS_1-2_ | H246R | L84P | 1.54 | 1.44 | 0.00 | 0.00 |
| M26 | HIS_1-2_ | L233V | --- | 0.00 | 0.00 | 0.64 | 0.16 |
| M27 | HIS_1-2_ | A217T | --- | 0.10 | 2.05 | 1.43 | 0.20 |
| M28 | HIS_3_ | --- | --- | 0.00 | 0.00 | 0.85 | 0.23 |
| M29 | HIS_3_ | --- | --- | 0.00 | 0.00 | 0.13 | 0.12 |
| M30 | HIS_3_ | --- | --- | 0.00 | 0.00 | 0.59 | 0.26 |
| M31 | HIS_3_ | V110D | --- | 0.00 | 3.45 | 0.91 | 0.02 |
| M32 | HIS_3_ | V110V Y139C | --- | 0.00 | 0.00 | 0.41 | 0.33 |
| M33 | HIS_3_ | A129A | --- | 0.00 | 0.00 | 0.56 | 0.23 |
| M34 | HIS_3_ | --- | L266P | 1.37 | 1.43 | 0.00 | 0.00 |
| M35 | HIS_3_ | --- | R315H | 1.38 | 1.41 | 0.07 | 0.02 |
| M36 | HIS_3_ | --- | --- | 0.00 | 0.00 | 0.12 | 0.10 |
| M37 | HIS_3_ | --- | --- | 0.00 | 0.00 | 0.55 | 0.27 |
| M38 | HIS_3_ | --- | --- | 0.00 | 4.77 | 1.04 | 0.01 |
| M39 | HIS_3_ | --- | Insertion of A at 5bp of P_tuf_ | 0.10 | 2.60 | 1.41 | 0.13 |
| M40 | HIS_3_ | --- | --- | 0.10 | 1.94 | 1.64 | 0.22 |
| M41 | HIS_1_ | --- | --- | 0.00 | 1.93 | 0.50 | 0.44 |
| M42 | HIS_1_ | L92L | W308 STOP | 0.00 | 3.97 | 0.00 | 0.00 |
| M43 | HIS_1-2_ | --- | Insertion of A at 74bp of P_tuf_ | 0.24 | 1.60 | 0.63 | 0.15 |
| M44 | HIS_1-2_ | --- | --- | 0.58 | 0.00 | 0.30 | 0.50 |
| M45 | HIS_1-2_ | --- | Insertion of A at 5bp of P_tuf_ | 0.30 | 1.64 | 0.62 | 0.13 |
| M46 | HIS_1-2_ | --- | Insertion of A at 5bp of P_tuf_ | 0.48 | 2.12 | 0.70 | 0.15 |
| M47 | HIS_1-2_ | A115V A132A | Insertion of A at 5bp of P_tuf_ | 0.39 | 2.38 | 0.90 | 0.10 |
| M48 | HIS_1-2_ | H123R V184M | --- | 0.00 | 3.34 | 0.00 | 0.00 |
| M49 | HIS_3_ | --- | Insertion of A at 5bp of P_tuf_ | 0.35 | 1.87 | 1.06 | 0.14 |
| M50 | HIS_3_ | --- | --- | 0.29 | 2.25 | 1.34 | 0.15 |
| M51 | HIS_3_ | S224P E244G | Insertion of A at 5bp of P_tuf_ | 0.04 | 3.42 | 0.00 | 0.00 |
| M52 | HIS_3_ | H246R | L84P | 1.54 | 1.27 | 0.00 | 0.00 |
| M53 | HIS_3_ | --- | --- | 0.94 | 0.97 | 0.82 | 0.14 |
| M54 | HIS_1-2_ | A115V | N/A | 0.46 | 0.68 | 0.44 | 0.30 |

Table S5: Dataset of means and standard deviations for quantified carotenoids [mg g_CDW_^-1^] and calculated cell dry weights (CDW) [g L^-1^] from technical triplicates corresponding to Figure 8.

Values below the detection limit were marked “N/A”.

| ID | CrtW_Fp_ | CrtZ_Fp_ | CDW | Lycopene | β-carotene | Echinenone | Canthaxanthin | Hydroxyechinenone | β-cryptoxanthin | Zeaxanthin | Adonirubin | Astaxanthin | Total carotenoids |
| --- | --- | --- | --- | --- | --- | --- | --- | --- | --- | --- | --- | --- | --- |
| BETA4 | --- | --- | 11.97 ±0.16 | N/A | 13.82 ±0.05 | N/A | N/A | N/A | N/A | N/A | N/A | N/A | 13.82 ±0.05 |
| M1 | D86G | --- | 11.78 ±0.58 | N/A | 14.99 ±1.20 | N/A | N/A | N/A | N/A | N/A | N/A | N/A | 14.99 ±1.20 |
| M24 | H246R | --- | 9.58 ±0.84 | N/A | 17.17 ±0.64 | N/A | N/A | N/A | N/A | N/A | N/A | N/A | 17.17 ±0.64 |
| M48 | H123R V184M | --- | 9.27 ±0.46 | N/A | 17.76 ±0.68 | N/A | N/A | N/A | N/A | N/A | N/A | N/A | 17.76 ±0.68 |
| M51 | S224P E244G | --- | 11.32 ±1.29 | N/A | 15.50 ±1.28 | N/A | N/A | N/A | N/A | N/A | N/A | N/A | 15.50 ±1.28 |
| M23 | S192P | --- | 10.38 ±0.98 | N/A | 15.59 ±1.63 | 0.39 ±0.12 | N/A | N/A | N/A | N/A | N/A | N/A | 15.99 ±1.61 |
| M31 | V110D | --- | 9.67 ±0.87 | N/A | 14.50 ±1.19 | 0.53 ±0.19 | 0.01 ±0.01 | N/A | N/A | N/A | N/A | N/A | 15.03 ±1.09 |
| M26 | L233V | --- | 9.83 ±1.12 | N/A | 12.11 ±2.65 | 0.97 ±0.07 | 0.06 ±0.01 | N/A | N/A | N/A | N/A | N/A | 13.13 ±2.69 |
| M27 | A217T | --- | 11.22 ±0.72 | N/A | 9.90 ±1.19 | 0.99 ±0.01 | 0.08 ±0.01 | N/A | N/A | N/A | N/A | N/A | 10.97 ±1.19 |
| M54 | A115V | --- | 10.60 ±0.23 | N/A | 8.93 ±1.60 | 1.48 ±0.06 | 0.11 ±0.05 | N/A | N/A | N/A | N/A | N/A | 10.52 ±1.50 |
| BETA4 *crtW_Fp_* | REF | --- | 8.57 ±0.65 | 0.11 ±0.02 | 6.88 ±1.92 | 1.94 ±0.15 | 0.38 ±0.19 | N/A | N/A | N/A | N/A | N/A | 9.31 ±1.66 |

Table S6: Dataset of means and standard deviations for quantified carotenoids [mg g_CDW_^-1^] and calculated cell dry weights (CDW) [g L^-1^] from technical triplicates corresponding to Figure 9.

Values below the detection limit were marked “N/A”.

| ID | CrtW_Fp_ | CrtZ_Fp_ | CDW | Lycopene | β-carotene | Echinenone | Canthaxanthin | Hydroxyechinenone | β-cryptoxanthin | Zeaxanthin | Adonirubin | Astaxanthin | Total carotenoids |
| --- | --- | --- | --- | --- | --- | --- | --- | --- | --- | --- | --- | --- | --- |
| BETA4 *crtZ_Fp_* | --- | REF | 7.82 ±0.69 | N/A | 13.10 ±0.57 | N/A | N/A | N/A | 0.34 ±0.04 | 1.40 ±0.21 | N/A | N/A | 14.83 ±0.79 |
| M1 *crtZ_Fp_* | D86G | REF | 6.32 ±0.98 | N/A | 10.34 ±2.47 | N/A | N/A | N/A | 0.28 ±0.10 | 1.30 ±0.30 | N/A | N/A | 11.91 ±2.85 |
| M24 *crtZ_Fp_* | H246R | REF | 8.50 ±1.52 | N/A | 12.87 ±1.53 | N/A | N/A | N/A | 0.33 ±0.09 | 1.01 ±0.31 | N/A | N/A | 14.20 ±1.90 |
| M48 *crtZ_Fp_* | S224P E244G | REF | 6.15 ±0.16 | N/A | 16.88 ±2.30 | N/A | N/A | N/A | 0.49 ±0.05 | 1.40 ±0.34 | N/A | N/A | 18.76 ±1.91 |
| M51 *crtZ_Fp_* | H123R V184M | REF | 6.98 ±1.08 | N/A | 14.97 ±1.50 | N/A | N/A | N/A | 0.42 ±0.11 | 1.26 ±0.45 | N/A | N/A | 16.65 ±1.45 |
| M31 *crtZ_Fp_* | V110D | REF | 9.20 ±0.12 | N/A | 10.53 ±0.85 | 0.06 ±0.01 | N/A | 0.06 ±0.01 | 0.26 ±0.01 | 0.77 ±0.05 | N/A | 0.18 ±0.03 | 11.87 ±0.75 |
| M23 *crtZ_Fp_* | S192P | REF | 9.28 ±0.59 | N/A | 10.54 ±0.64 | 0.03 ±0.01 | N/A | 0.04 ±0.01 | 0.26 ±0.02 | 0.84 ±0.06 | N/A | 0.23 ±0.03 | 11.93 ±0.76 |
| M27 *crtZ_Fp_* | A217T | REF | 8.68 ±1.16 | 0.15 ±0.06 | 4.00 ±0.79 | 0.27 ±0.06 | 0.02 ±0.00 | 0.21 ±0.01 | 0.15 ±0.03 | 1.05 ±0.24 | N/A | 1.06 ±0.26 | 6.91 ±0.79 |
| BETA4 *crtZ_Fp_* *crtW_Fp_* | REF | REF | 8.92 ±0.12 | 0.10 ±0.02 | 4.64 ±0.82 | 0.33 ±0.02 | 0.03 ±0.01 | 0.30 ±0.02 | 0.12 ±0.02 | 0.66 ±0.05 | N/A | 1.17 ±0.14 | 7.36 ±0.67 |
| M26 *crtZ_Fp_* | L233V | REF | 6.58 ±0.15 | 0.25 ±0.01 | 4.34 ±0.16 | 0.21 ±0.01 | 0.01 ±0.00 | 0.17 ±0.01 | 0.20 ±0.01 | 1.77 ±0.06 | N/A | 1.21 ±0.03 | 8.15 ±0.15 |
| M54 *crtZ_Fp_* | A115V | REF | 10.12 ±0.45 | 0.10 ±0.04 | 4.30 ±1.60 | 0.28 ±0.03 | 0.01 ±0.01 | 0.29 ±0.02 | 0.10 ±0.04 | 0.60 ±0.05 | N/A | 1.25 ±0.39 | 6.94 ±1.20 |
